# Supplementary material for: Conditional cash transfer program and child mortality: A cross-sectional analysis nested within the 100 Million Brazilian Cohort
Source: PLoS Med. 2021 Sep 28;18(9):e1003509. doi: 10.1371/journal.pmed.1003509 (PMC8478244; doi:10.1371/journal.pmed.1003509)
Supplement: S1 Checklist — REporting of studies Conducted using Observational Routinely-collected health Data (RECORD) guideline. (DOCX) [file pmed.1003509.s001.docx]

**The RECORD statement – checklist of items, extended from the STROBE statement, that should be reported in observational studies using routinely collected health data.**

|  | **Item No.** | **STROBE items** | **Location in manuscript where items are reported** | **RECORD items** | **Location in manuscript where items are reported** |
| --- | --- | --- | --- | --- | --- |
| **Title and abstract** | | | | | |
|  | 1 | (a) Indicate the study’s design with a commonly used term in the title or the abstract (b) Provide in the abstract an informative and balanced summary of what was done and what was found | Title page | RECORD 1.1: The type of data used should be specified in the title or abstract. When possible, the name of the databases used should be included.  RECORD 1.2: If applicable, the geographic region and timeframe within which the study took place should be reported in the title or abstract.  RECORD 1.3: If linkage between databases was conducted for the study, this should be clearly stated in the title or abstract. | Title page |
| **Introduction** | | | | | |
| Background rationale | 2 | Explain the scientific background and rationale for the investigation being reported | pages 3 and 4 |  | Introduction |
| Objectives | 3 | State specific objectives, including any prespecified hypotheses | Introduction, last paragraph |  | Introduction, last paragraph |
| **Methods** | | | | | |
| Study Design | 4 | Present key elements of study design early in the paper | Methods section, first paragraph |  | Methods section, first paragraph |
| Setting | 5 | Describe the setting, locations, and relevant dates, including periods of recruitment, exposure, follow-up, and data collection | Methods section, 5th paragraph (Population definition) |  | Methods section, 5th paragraph (Population definition) |
| Participants | 6 | *(a) Cohort study* - Give the eligibility criteria, and the sources and methods of selection of participants. Describe methods of follow-up  *Case-control study* - Give the eligibility criteria, and the sources and methods of case ascertainment and control selection. Give the rationale for the choice of cases and controls  *Cross-sectional study* - Give the eligibility criteria, and the sources and methods of selection of participants  *(b) Cohort study* - For matched studies, give matching criteria and number of exposed and unexposed  *Case-control study* - For matched studies, give matching criteria and the number of controls per case | Methods section, 5th paragraph (Population definition); Figure 1 | RECORD 6.1: The methods of study population selection (such as codes or algorithms used to identify subjects) should be listed in detail. If this is not possible, an explanation should be provided.  RECORD 6.2: Any validation studies of the codes or algorithms used to select the population should be referenced. If validation was conducted for this study and not published elsewhere, detailed methods and results should be provided.  RECORD 6.3: If the study involved linkage of databases, consider use of a flow diagram or other graphical display to demonstrate the data linkage process, including the number of individuals with linked data at each stage. | Record 6.1 - Methods section, 5th paragraph (Population definition); Figure 1  Record 6.3 - Methods section (3rd and 4th paragraphs, Data Linkage); Figure S1a |
| Variables | 7 | Clearly define all outcomes, exposures, predictors, potential confounders, and effect modifiers. Give diagnostic criteria, if applicable. | Methods section (6th-9th and 13th-15th paragraphs), | RECORD 7.1: A complete list of codes and algorithms used to classify exposures, outcomes, confounders, and effect modifiers should be provided. If these cannot be reported, an explanation should be provided. | Methods section (6th-9th and 13th-15th paragraphs) |
| Data sources/ measurement | 8 | For each variable of interest, give sources of data and details of methods of assessment (measurement).  Describe comparability of assessment methods if there is more than one group | Methods section (1st and 2nd paragraphs, Data description) |  | Methods section (1st and 2nd paragraphs, Data description) |
| Bias | 9 | Describe any efforts to address potential sources of bias | Methods section (8th-12th paragraphs) |  | Methods section (8th-12th paragraphs) |
| Study size | 10 | Explain how the study size was arrived at | Figure 1  Results section (1st paragraph) |  | Figure 1  Results section (1st paragraph) |
| Quantitative variables | 11 | Explain how quantitative variables were handled in the analyses. If applicable, describe which groupings were chosen, and why | Quantitative variables involved in the analysis were: registration year, maternal age (categorized in three groups), Municipal HDI and DMI (in quintiles). Information regarding these variables can be found throughout the methods section. |  | Quantitative variables involved in the analysis were: registration year, maternal age (categorized in three groups), Municipal HDI and DMI (in quintiles). Information regarding these variables can be found throughout the methods section. |
| Statistical methods | 12 | (a) Describe all statistical methods, including those used to control for confounding  (b) Describe any methods used to examine subgroups and interactions  (c) Explain how missing data were addressed  (d) *Cohort study* - If applicable, explain how loss to follow-up was addressed  *Case-control study* - If applicable, explain how matching of cases and controls was addressed  *Cross-sectional study* - If applicable, describe analytical methods taking account of sampling strategy  (e) Describe any sensitivity analyses | Methods section (8th to last paragraphs) |  | Methods section (8th to last paragraphs) |
| Data access and cleaning methods |  | .. |  | RECORD 12.1: Authors should describe the extent to which the investigators had access to the database population used to create the study population.  RECORD 12.2: Authors should provide information on the data cleaning methods used in the study. | Methods section (5th paragraph, Population definition);  Figure 1 |
| Linkage |  | .. |  | RECORD 12.3: State whether the study included person-level, institutional-level, or other data linkage across two or more databases. The methods of linkage and methods of linkage quality evaluation should be provided. | Methods section (3rd and 4th paragraphs - Data Linkage);  Supporting Information 1 (Data Linkage) |
| **Results** | | | | | |
| Participants | 13 | (a) Report the numbers of individuals at each stage of the study (*e.g.*, numbers potentially eligible, examined for eligibility, confirmed eligible, included in the study, completing follow-up, and analysed)  (b) Give reasons for non-participation at each stage.  (c) Consider use of a flow diagram | Results section (1st paragraph);  Figure 1 | RECORD 13.1: Describe in detail the selection of the persons included in the study (*i.e.,* study population selection) including filtering based on data quality, data availability and linkage. The selection of included persons can be described in the text and/or by means of the study flow diagram. | Results section(1st paragraph);  Figure 1 |
| Descriptive data | 14 | (a) Give characteristics of study participants (*e.g.*, demographic, clinical, social) and information on exposures and potential confounders  (b) Indicate the number of participants with missing data for each variable of interest  (c) *Cohort study* - summarise follow-up time (*e.g.*, average and total amount) | Table 1 |  | Table 1 |
| Outcome data | 15 | *Cohort study* - Report numbers of outcome events or summary measures over time  *Case-control study* - Report numbers in each exposure category, or summary measures of exposure  *Cross-sectional study* - Report numbers of outcome events or summary measures | Results section (1st paragraph) |  | Results section (1st paragraph) |
| Main results | 16 | (a) Give unadjusted estimates and, if applicable, confounder-adjusted estimates and their precision (e.g., 95% confidence interval). Make clear which confounders were adjusted for and why they were included  (b) Report category boundaries when continuous variables were categorized  (c) If relevant, consider translating estimates of relative risk into absolute risk for a meaningful time period | Tables 2-6  Tables |  | Tables 2-6 |
| Other analyses | 17 | Report other analyses done—e.g., analyses of subgroups and interactions, and sensitivity analyses | Results section (2nd until last paragraphs) and Tables 2-6;  Table S1c  Supporting Information 2 |  | Results section (2nd until last paragraphs) and Tables 2-6;  Table S1c  Supporting Information 2 |
| **Discussion** | | | | | |
| Key results | 18 | Summarise key results with reference to study objectives | Discussion section (1st paragraph) |  |  |
| Limitations | 19 | Discuss limitations of the study, taking into account sources of potential bias or imprecision. Discuss both direction and magnitude of any potential bias | Discussion, Strengths and Limitations section | RECORD 19.1: Discuss the implications of using data that were not created or collected to answer the specific research question(s). Include discussion of misclassification bias, unmeasured confounding, missing data, and changing eligibility over time, as they pertain to the study being reported. | Discussion, Strengths and Limitations section |
| Interpretation | 20 | Give a cautious overall interpretation of results considering objectives, limitations, multiplicity of analyses, results from similar studies, and other relevant evidence | Discussion (1st-8th paragraphs) |  | Discussion (1st-8th paragraphs) |
| Generalisability | 21 | Discuss the generalisability (external validity) of the study results |  |  |  |
| **Other Information** | | | | | |
| Funding | 22 | Give the source of funding and the role of the funders for the present study and, if applicable, for the original study on which the present article is based | This work was supported by the Grand Challenges Brazil grant MCTI/CNPq/MS/SCTIE/Decit/Fundação Bill and Melinda Gates call Nº 47/2014 and the Wellcome Trust (201912/B/16). The funding agencies had no role in the design and conduct of the study; collection, analysis, and interpretation of the data; or the writing of the manuscript or the decision to submit it for publication. |  | This work was supported by the Grand Challenges Brazil grant MCTI/CNPq/MS/SCTIE/Decit/Fundação Bill and Melinda Gates call Nº 47/2014 and the Wellcome Trust (201912/B/16). The funding agencies had no role in the design and conduct of the study; collection, analysis, and interpretation of the data; or the writing of the manuscript or the decision to submit it for publication. |
| Accessibility of protocol, raw data, and programming code |  | .. | Data accessibility statement | RECORD 22.1: Authors should provide information on how to access any supplemental information such as the study protocol, raw data, or programming code. | Data accessibility statement |

*Reference: Benchimol EI, Smeeth L, Guttmann A, Harron K, Moher D, Petersen I, Sørensen HT, von Elm E, Langan SM, the RECORD Working Committee. The REporting of studies Conducted using Observational Routinely-collected health Data (RECORD) Statement. *PLoS Medicine* 2015; in press.

*Checklist is protected under Creative Commons Attribution ([CC BY](http://creativecommons.org/licenses/by/4.0/)) license.
