# Supplementary figures and images for: Conditional cash transfer program and child mortality: A cross-sectional analysis nested within the 100 Million Brazilian Cohort
Source: PLoS Med. 2021 Sep 28;18(9):e1003509. doi: 10.1371/journal.pmed.1003509 (PMC8478244; doi:10.1371/journal.pmed.1003509)

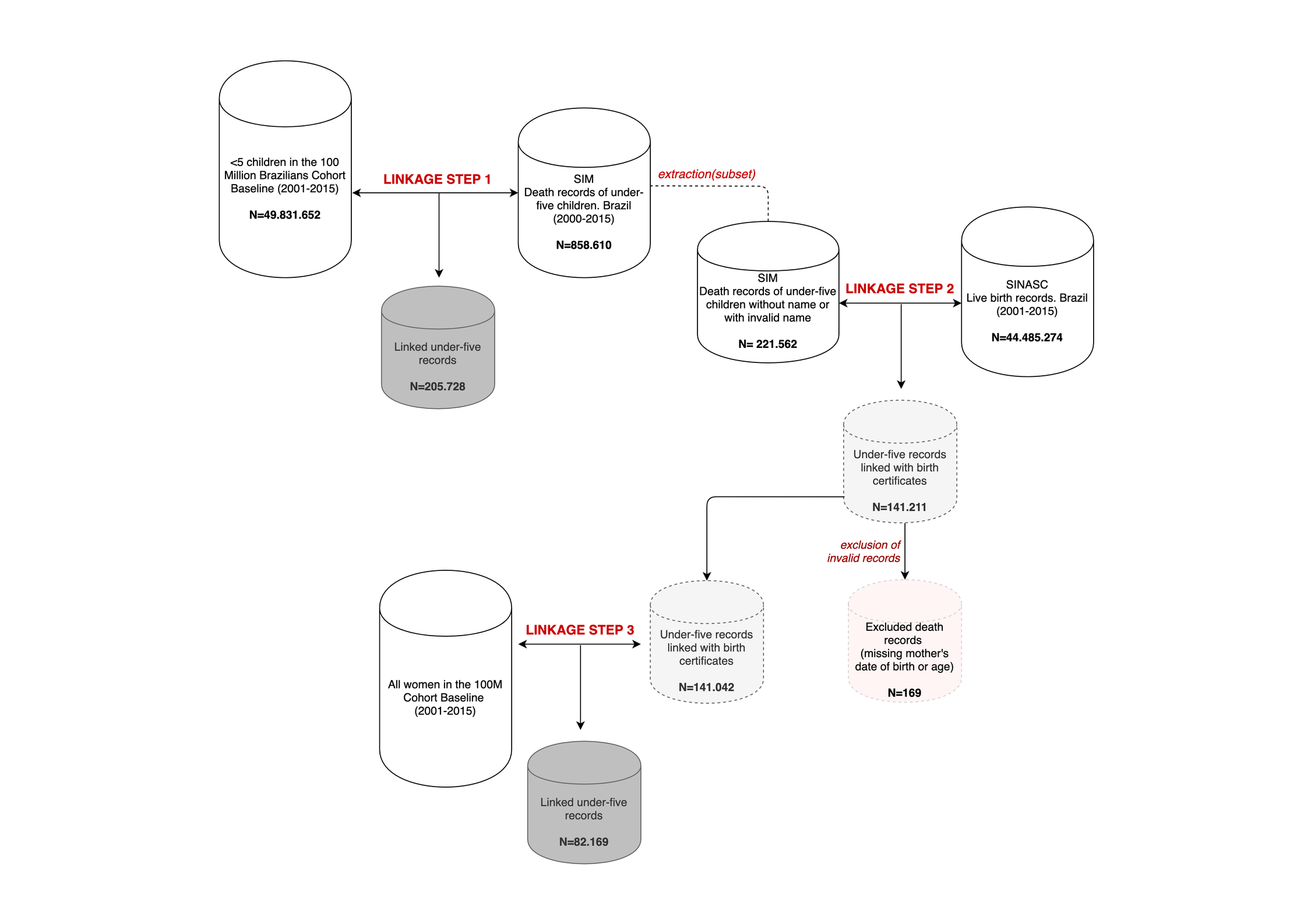

Supplement: S1 Fig — Flowchart of the linkage process between the CIDACS 100 Million Brazilian Cohort baseline dataset, mortality data (Brazilian Mortality Information System [SIM]), and live birth records (Brazilian Live Birth Information System [SINASC]). (TIF) [file pmed.1003509.s002.tif]

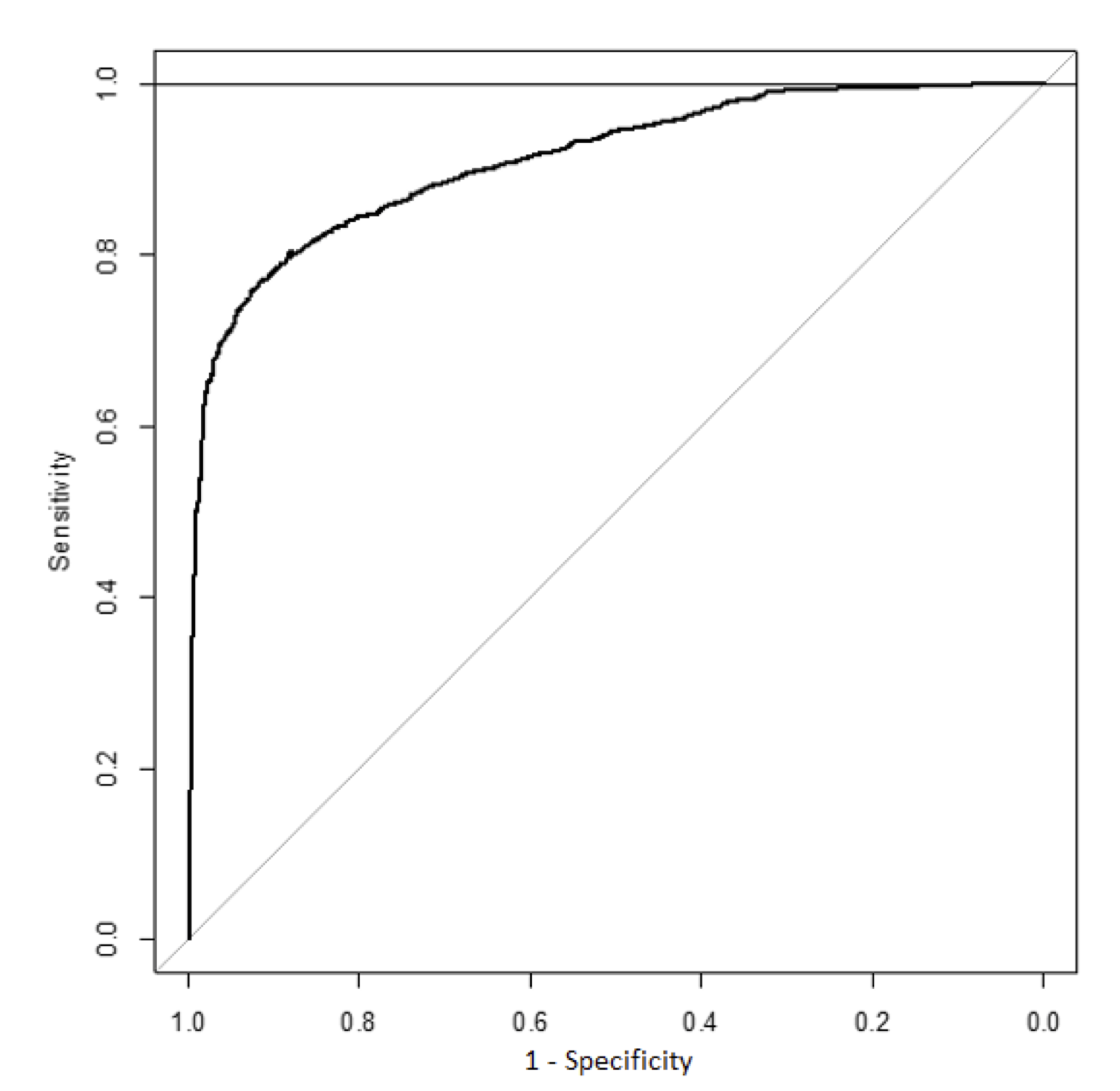

Supplement: S2 Fig — (TIF) [file pmed.1003509.s003.tif]

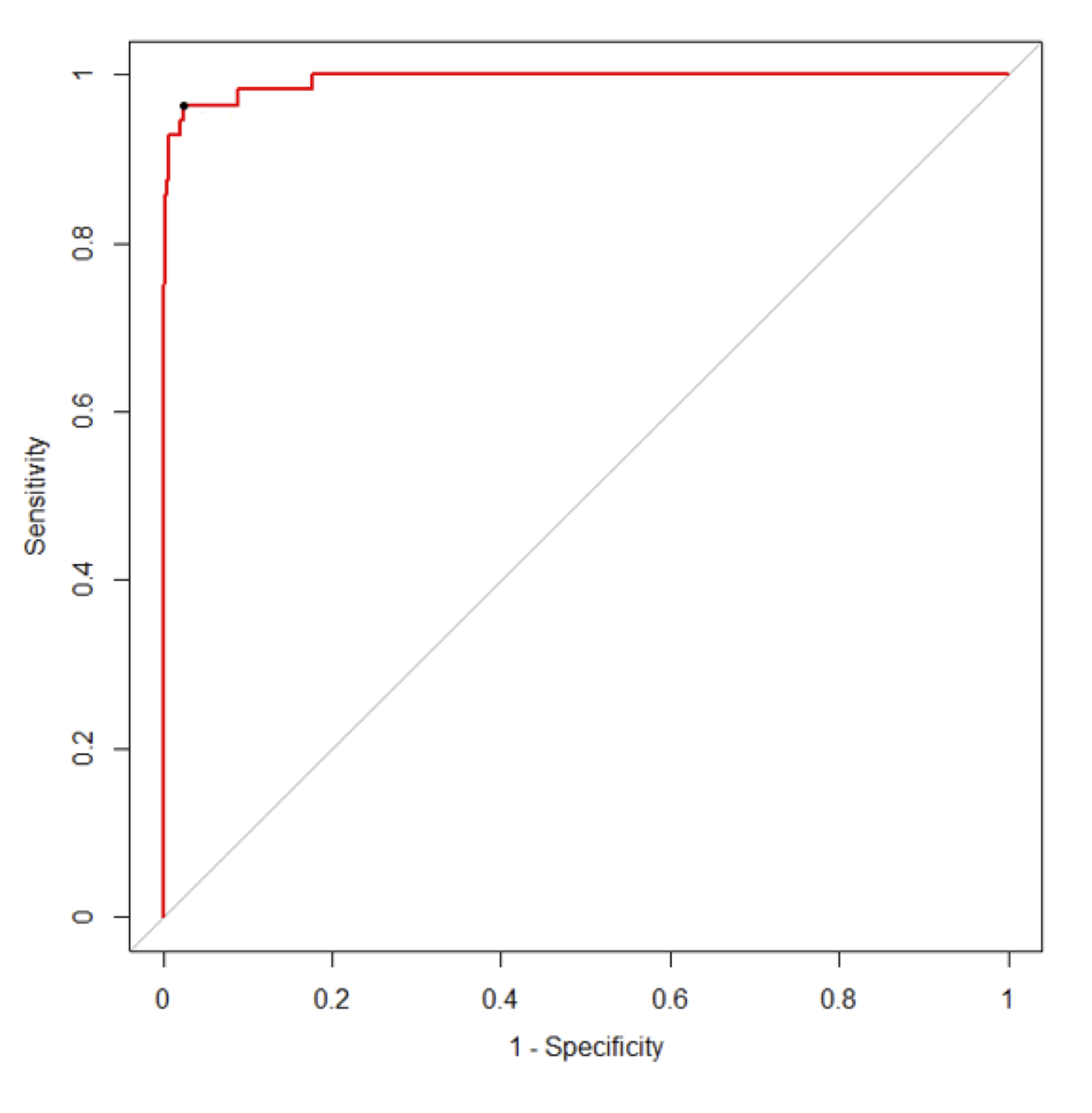

Supplement: S3 Fig — (TIF) [file pmed.1003509.s004.tif]

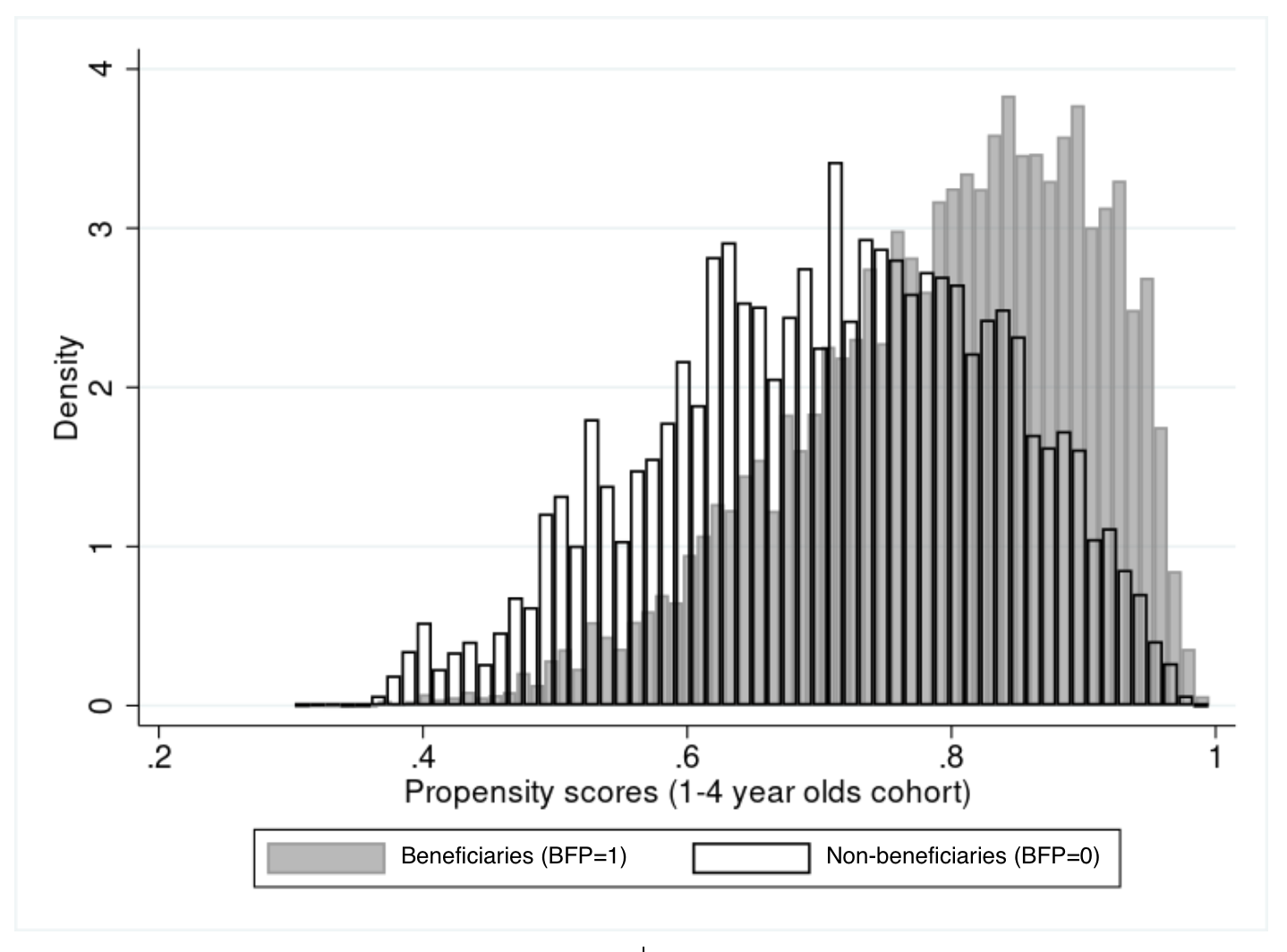

Supplement: S4 Fig — Distribution of propensity scores across beneficiaries and non-beneficiaries. (TIF) [file pmed.1003509.s005.tif]
