# Supplementary material for: Conditional cash transfer program and child mortality: A cross-sectional analysis nested within the 100 Million Brazilian Cohort
Source: PLoS Med. 2021 Sep 28;18(9):e1003509. doi: 10.1371/journal.pmed.1003509 (PMC8478244; doi:10.1371/journal.pmed.1003509)
Supplement: S1 Text — (PDF) [file pmed.1003509.s006.pdf]

Anexo I (versão em inglês)

CHAMADA MCTI/CNPq/MS/SCTIE/Decit/Fundação Bill e Melinda Gates N º 47/2014

Grand Challenge Brazil
Application Form and Instructions

Submission Date:

Proposal Information

A. Organization

Organization Name: Fundação Oswaldo Cruz – FIOCRUZ

Institutional Official authorized to submit and accept grants on behalf of organization:

Prefix	*Dr.*	*First name*	*Manoel*	Surname	*Barral-Netto*	*Suffix*
Title	Professor			*Telephone*		
	Centro de Pesquisas Gonçalo Moniz - CPqGM.<br>Rua Waldemar Falcão, 121, Candeal - Salvador/BA CEP: 40296-710			*Fax*		
E-mail						
Web site	http://www.bahia.fiocruz.br/					

B. Project

Project Name: Evaluating the impact of social factors and interventions on healthy growth and development: the 100 million Brazilian cohort

Principal Investigator/Project Director:

Prefix	*Dr.*	*First name*	*Mauricio*	Surname	*Barreto*	*Suffix*
Title	Professor			*Telephone*		
Address	Centro de Pesquisas Gonçalo Moniz - CPqGM.<br>Rua Waldemar Falcão, 121, Candeal - Salvador/BA CEP: 40296-710			*Fax*		
E-mail						
Web site	http://www.bahia.fiocruz.br/					

Amount Requested ($USD): $ 1,310,880.98

Project Duration (months): 48 months

Estimated Total Cost of Project ($USD): $1,970,000

Organization's total revenue for most recent audited financial year ($USD): $885,000,000 $

I. Main Goal

The project, based on the Brazilian 100 Million cohort and datacenter (linking data from social security records (Cadastro Único - CADU), to routine records on births, deaths, and grown monitoring), will produce strong and specific evidence of the impact of social interventions and social deprivation on still births, prematurity, low birthweight, infant mortality (by cause and age) and growth in the first years of life, nationally and by subgroups (e.g., by education of mother) and timing of intervention (e.g., preconception, early or late pregnancy). The evidence will strengthen the case for implementation of social interventions – in addition to biomedical interventions - to promote healthy birth, child health and development. A legacy of the project will be the cohort and plans for a public interface for the cohort and datacenter, a facility for production of anonymized datasets to support other researchers and public managers, for methodological development, training and capacity development.

II. Executive Summary

Context The last two decades witnessed major social changes in Brazil, involving the economy (e.g., minimum wage increasing above inflation), the health sector (with a new primary health care program-Family Health Strategy reaching high coverage and equality in access) and health related measures from other sectors (i.e., improvement in water and sanitation, education) with a parallel decrease in the level of social inequalities. During the same period the country has undergone substantial health changes, including a large reduction in under nutrition (now below 5%) and in deaths of children under 5 years of age. The proposed Brazilian 100 Million cohort and datacenter is an unprecedented and innovative resource which aims to generate knowledge to be used in evaluative research initially on health birth, growth and development to contribute to the understanding of factors behind the health changes observed and to inform decision making. To support the social programs Brazil has developed a central database (Cadastro Único - CADSU) registering the information collected from all potential candidates to receive conditional cash transfer or other social programs implemented by the Brazilian Federal Government. The cohort will based on this existing large data base (with over 100 million individuals) to be linked to several rich and robust routine data sets of health and other potential outcomes. The unique size of the dataset and its longitudinal characteristic will allow investigation of impact of environment and interventions received in different moments of life (in utero, different stages of pregnancy, early or late childhood) and in different subpopulation defined for example by geography, income, gender, and environment. **Project goal, objectives, and critical milestones** The overall objective of the project is to conduct research on effect of social determinants and impact of social policies on healthy birth, growth and survival overall and in subgroups of interest in a virtual cohort of 100 million Brazilian originated from CADU records since 2007. We plan to establish in a datacenter organized around safe linkage of data from the national conditional cash transfer Program (Bolsa Familia, BF) including BF monthly payments and conditionalities, with birth notifications (SINASC), death certificates (SIM) and growth monitoring. Later we will plan the development of a sustainable public interface managing access to researchers and policy makers to anonymized datasets from this depository, offering methodological development, training and capacity strengthening resources. The datacenter aims to establish standards of governance and ethics, including protection of privacy. The overall goal will be achieved by four specific objectives: 1) To setup the 100 million Brazilians cohort and data center; 2) To estimate the effect of social and economic factors, antenatal care and maternal characteristics on birth weight, prematurity, fetal death, date and cause of death in the five year of life, and growth in the first 5 years of life overall and in sub populations and periods; 3) To estimate the impact of Bolsa Familia on birth weight, prematurity, fetal death, mortality by age and cause and on growth on children in the first five years of life, overall and in sub populations and periods; 4) To set up a strategic plan for the future of the data center including financial sustainability and plans for developing a public interface for the datacenter as a resource for researchers and decision makers. The strategic vision is to expand the cohort to include all ages, additional health data (infections, hospitalizations, cancer registrations) and eventually to educational performance (e.g., Nation Examination of Secundary Education - ENEM) and social and economic performance (employment, divorce etc). The critical Milestones are 1a) Ethical clearance granted and the five datasets (CADU, BF payments, SINASC (births), SIM (mortality including still births), and GM- growth monitoring) in our possession; 1b) Functioning datacenter, with adequate space, necessary computing facilities, tested software, final Standardized Operational Procedures -SOPs for linkage, data cleaning and validation and data extraction; 1c- Cohort ready for data analysis; 2a- Preparation of plan of analysis identifying all specific research questions to be analyzed and detailing the methods to be used; 2b- Perform analysis of data, interpretation and write up results; 2c -Submission of the first 2 papers in

high impact journals; 2d- Dissemination of findings with policy implications to policy makers; 3a- Preparation of plan of analysis identifying all specific research questions to be analyzed and detailing the methods to be used; 3b- Submission of the first 2 papers in high impact journals; 3c- Dissemination of findings with policy implications to policy makers; 4a) A clearly defined plan for the continuation and expansion of the cohort and data center; 4b) A detailed plan for the implementation of the public interface; 4c) A clearly defined strategic plan for long term funding and sustainability. **Organizational capacity/management plan** The main institutions involved (Fundação Oswaldo Cruz, Instituto de Saude Coletiva/Universidade Federal da Bahia e London School of Hygiene and Tropical Medicine are recognized institutions in the field of public health research with international reputation for excellence. The team has undisputable experience in the conduction of large and complex studies and a track record of high impact publications. The project will be led by the PI, Mauricio Barreto and a small executive committee, consisting of the LSHTM PI, Laura Cunha Rodrigues, and the coordinators of three working groups (IT, Research and Cohort Administration), with all staff responding to one of the coordinators. This will be supported by a Scientific Committee which includes all co-PIs and an Advisory Committee to be appointed, but which will include the two collaborators, Prof Joy Lawn, from LSHTM (MARCH) and from Saving Newborn Lives and Prof Liam Smeeth from the LSHTM and the Farr Institute.

III. Context

Large social inequalities and poverty are major historical characteristics of Brazil. There is accumulated evidence that disruption (being biological or social) during the first years of life have important consequences creating conditions for increased risks of disease (cardiovascular, diabetes, depression etc) later in life.(1). While the gap among poor and rich countries is large (2), a recent review summarizes the evidence of the impact of inequalities on healthy birth and development in UK (3): inequalities influence health and well-being of the mother, the intra-uterine environment and the foetal development before birth, increasing the risk of foetal death as well as of low birth weight and prematurity. The report and other evidence also reminds us that negative birth outcomes, such low birthweight, not only influence mortality and development during early years and school-readiness but also educational attainment, economic participation and long-term health outcomes. The WHO's 2008 report, followed by the World Conference on Social Determinants on Health in 2011 gave great emphasis on the role of health child development to decrease present and future disparities in health (1).

In the past two decades, Brazil has undergone substantial health changes, including a large reduction in under nutrition (now below 5%) and in deaths of children under 5 years of age. These rapid improvements enabled the country to reach the fourth Millennium Development Goal even before the 2015 deadline.(4,5,6) This is a timely period to investigate the role of social differentials, and the impact of social interventions, on healthy birth, growth and development.

The last two decades witnessed major social changes in Brazil, involving the economy (e.g, minimum wage increasing above inflation), the health sector (with an audacious primary health care program-Family Health Strategy or FHS reaching high coverage and great equality in access (6,7,8) and health related measures from other sectors (improvement in water and sanitation, education etc) with a parallel, important decrease and in the level of social inequalities. We propose that a specific social intervention – the Brazilian cash transfer program, Bolsa Familia (PBF), contributed to these health achievements (9,10). The Brazilian experience appears to show that a strategic combination of health and social programs can increase access, reduce health inequities and under nutrition and improve health outcomes.

Cash transfer programs have lately been implemented in many countries as part of the policies to overcome poverty and social inequality (Figure 1). They consist of direct transfer of money to eligible people; sometimes they impose conditions (conditional cash transfer programs, CCT). Frequently conditions are related to education and health. The transfer of benefits aims to promptly alleviate poverty and the conditions encourage use of existing health and education services.(11,12)

Figure 1 -Countries with Cash Transfer Programs(in blue)

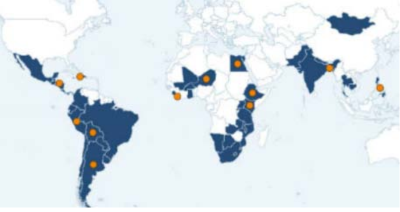

In Brazil, the *Bolsa Familia* program (Family Allowance, BFP) started in 2003 as result of the amalgamation of four preexisting national social programs. The BFP is the world's largest CCT program, and its coverage has expanded and consolidated in the past 12 years. It reached all of the nearly 6000 Brazilian municipalities and enrolled over 13 million families (13). Its total yearly budget is over US$11 billion.(4) BFP is intended for (a) "extremely poor" families (with an income of less than $35 per person per month) and (b) other families deemed poor, with an income of between $35-$70 per person per month when they include children up to 17 years of age or pregnant or breastfeeding women. Benefits per family can range from $18 to $175 per month, related to the level of poverty and the presence of pregnant women, children, or adolescents. Payment are always given to a woman (when present) on behalf of the whole family. BFP is a conditional cash transfer program: BFP family recipient must comply with specified education and health-related conditions. The health conditions are: a) children up to 7 years must receive all vaccines in accordance with the country immunization program schedule and must comply with health checks and growth monitoring according to Ministry of Health guidelines; b) pregnant and breastfeeding women must attend scheduled prenatal and postnatal visits and health and nutritional educational activities.

Evaluating the impact of cash transfer programs

Studies of impact of CCT programs, conducted in different countries (some summarized in reviews), have shown that CCTs can improve the use of health services, nutritional status and health outcomes of children (14,15). In Brazil, evaluations of PBF have found evidence of a positive effect of the Brazilian cash-transfer program (Bolsa Familia - PBF) on food acquisition by the family, nutritional status and health outcomes (16,17). A recent study, conducted by our group (9) using panel data analysis of a large number of Brazilian municipalities for the period 2004-9 showed that municipalities with high PBF coverage (defined as the proportion of the municipal population below the poverty line receiving benefits from the Program) had significantly lower under-5 mortality, overall and from poverty-related causes such as malnutrition and diarrhea in that municipality. Similar finding was obtained in another study with a different approach (10). High municipal coverage of PBF also appeared to reduce substantially municipal rates of under-5 admission to hospital by the same causes and increase coverage of vaccination and prenatal visits in municipalities. Further studies from our group, also using panel data, have shown impact on municipal rates of incidence of leprosy (18) and tuberculosis (unpublished data). These findings are compatible with the high speed in which the Brazilian nutritional and health situation has changed in the past few years and compatible with the data accumulated for several decades on the role of social determinants on health in particular on child health and infectious diseases.

Empiric evaluation of the impact of policies and complex interventions is an extremely important source of robust evidence, and an evolving interdisciplinary field in conceptual and methodological development (19,20,21,22). The use of randomized trials as source of evidence is overstressed by some but relativized by others. RCTs can not be used to evaluate the impact of policies already implemented; but in addition, there are arguments that the randomized trial evaluates not the impact of a real policy but of a sanitized version of the policy, as trials are conducted under extremely controlled conditions. The alternative is to evaluate intervention in process by use of non randomized methods (generically denominated quasi-experimental studies). Quasi experimental evaluation is a modern and expanding field. The experience, and the methodological developments come from different disciplines (economy, social sciences, political sciences, epidemiology etc) and led to a series of approaches to ensure study robustness. The quasi experimental approach can not only approximate the analytical rigor of the classical randomized approach, but have the advantage of studying the real intervention as delivered in the ground.

An important requirement of the non-randomized evaluation is the availability of data with good, detailed measurement of the intervention, covariates and outcomes. The quality of administrative data in Brazil is constantly improving. Brazil has a long tradition of production of electronic data bases, and to perceive these data bases as important sources of knowledge through research, There is a large body of research evaluating the quality of routinely produced electronic data as well as substantive health research conducted using these data. Our group we have produced detailed impact evaluation of large national programs as FHS and BFP using secondary health data using ecological designs (7,8,9,18). The data to be used in the present study come from available in electronic data sources and all have been evaluated and shown to of acceptable level of completeness, accuracy.

To support the social programs Brazil has developed a central database (Cadastro Único-CADU) registering the information collected from all potential candidates to receive conditional cash transfer or other social programs implemented by the Brazilian Federal Government (Figure 2).

Figure 2 Social Programs using Cadastro Único

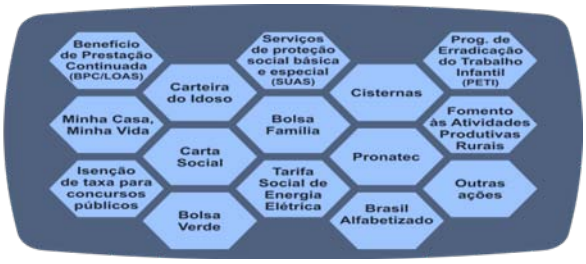

The 100 million cohort The Database is electronic, has detailed information on social, environmental, and economic features of over 100 million named individuals grouped into families; the information is renewed periodically as long as the person is a candidate to receive the program(23). Linking this database to the BF monthly payments (for those in CADU that receive it) and in sequence linking all the cohort with health records (i.e. births, mortality, nutritional status) will create a unique research resource on an unprecedented scale. The data to be used in this study include the cohort baseline data, that includes individual records from CADU, family monthly payments of BF, birth outcomes (still birth, prematurity, birth weight), mortality by cause and growth in the first five year of life.

The following data bases will be used in the present analysis, initially for the years 2007 to 2012;

1- Cadastro Unico (CADU) (It is an extensive questionnaire with information on the household and each individual. It include demographic, economic, social, and conditions of the household)

2- Monthly payment of Bolsa Familia (BF), includes the following variables: For each month, value of benefit received, if any;

3- Birth notifications (SINASC), includes the following variables: date of birth, place of delivery (hospital or home) and for the mothers: age, schooling, marital status, number of previous live and still births, and for the new born: gestational age, birthweight, type of pregnancy (singleton or twin) type of delivery, sex, ethinicity, congenital abnormality (presence and type) for live and still births; for live births, also apgar 1', 5'.

4- Death certificate (SIM) for foetal deaths and from 0-5 ys include the following variables: date of birth, date of death, sex, ethnicity, and where the death occurred (hospital, other type of health unit, at home, elsewhere) and ICD10 code causes of death.

5- Growth Monitoring (GM) for children from families receiving BF from 2007 to 2012. includes the following variables: weight height age (monthly for children under 7) vaccinations; antenatal care consultations

This unprecedented cohort will enable the investigation of the role of major social factors on nutritional and health outcomes, in a classical longitudinal observational approach, but will also enable to study in great detail the impact of BF on the same outcomes, as well as over a variety of strata never investigated (i.e by each level of education f mother or stage of pregnancy). The cohort and datacenter will also serve as a

center for methodological development, training and capacity building, and the plans for a public interface will be defined.

This will be the largest data source of linked health and social data ever assembled, offering unprecedented power to tackle unanswered questions of major importance: 100 million subjects followed for many years. The main comparative advantage of this approach is the sheer magnitude of the data, providing unprecedented power to investigate not only large but also small effects, variation of effect depending on when in the life course (at what period of preconception, gestation and childhood) are social interventions more effective, and exploration of effects variation across very different social environments in Brazil; additional advantages are the linkage, organizing and relating data from different, information rich sources from social and health data; the longitudinal approach, allowing investigation of temporality of associations; and the legacy: the digital, anonymized big data 100 million cohort and data center which will serve as a powerful unprecedented research resort for generations to come. This will make the most of the digital era, “big data” approaches to knowledge generation (24), and Brazil’s open, modern attitude towards making electronic routine data easily accessible to researchers. The datacenter will bring accessibility, confidentiality, ethical rigour, and standardization to this utilization. We believe there is a ethical mandate to make best research use of already collected data which has been shown to be of excellent quality

The extraordinary opportunities created by this unprecedented dataset open a variety of new possibilities. Firstly, it will shed new light on the historical debate about the impact of social policies on health. In developed countries much of the improvement in areas such as child health and infectious diseases happen before modern health technological resources. The understanding of the factors responsible for such changes are still an area of dispute, but is clear the great importance to social polices (25,26). The implementation of social policies in such a large scale as Bolsa Familia in Brazil creates a unique opportunity to review and contribute to this important historical question.

However, the most important contribution of the proposed research will be the generation of evidence of the impact of social determinants and social policies (in special a cash transfer program - BF) on to healthy birth, nutrition, survival and development It is known that integration of social policy as integral part of health policy requires solid, detailed evidence of impact, but very frequently this evidence not exist or it is not sufficient robust to justify policies. In particular, and very much novel, and relevant to efficient targeting of policies, the magnitude of the cohort will guarantee power to investigate these questions in a great deal of detail: how the impact of social policies, and health outcomes is different according to different aspects of the benefit and when it is received, and specific characteristics of the recipient .

Once the data center is established, it will be available to policy makers and researchers to answer a range of policy, managerial, and scientific questions in a short time, with very low cost, and with methodological rigour. The search for ways to integrate knowledge and practices, science and policies has a long and frequently unsuccessful past. The proposed common use of the resource by academicians and decision-makers will create a unique environment for interactions between them and in real time to create opportunities for rigorous scientific analysis to be used as evidence in policy making

IV. Project Framework

Project Framework Table

	**Results**	**Period of Activity**	**Approximate Total Cost**
**Overall objective**<br>To conduct research on social effect and impact of social policies on healthy birth, growth and survival overall and in subgroups of interest in an unprecedented virtual cohort of 100 million Brazilians originated from CADU subjects	Evidence on social effect and impact of social policies on healthy birth, growth and survival overall and in subgroups of interest<br><br>Virtual cohort 100 million Brazilian and datacenter with plans to develop a sustainable public interface	1-48 months	US$<br>1,310,880.98

included from 2007, in a properly established datacenter organized around safely linkage of CADU data with Bolsa Familia monthly payments, SINASC, SIM and growth monitoring from BF conditionalities, and to plan the development of aa sustainable public interface managing access to researchers and policy makers to this depository, offering methodological development, training and capacity building resources.	managing access to researchers and policy makers as below:		
**<u>Objective 1:</u>**<br>Setup of the 100 million Brazilians cohort and data center<br>.<br>,	A functioning datacenter with the cohort ready for analysis, including:<br>Ethical clearance granted and the five datasets (CADU, BF payments, SINASC, SIM, and GM) in our possession<br>Functioning datacenter, with adequate space, necessary computing facilities, tested software, final SOPs for linkage, data cleaning and validation and data extraction.<br>Cohort ready for data analysis. Paper on the cohort profile submitted;	0-18 months<br>0- 6 months.<br>0-12 months<br>6-18 months	US$<br>1,002,760.98
**Objective 2**:<br>To estimate the effect of social and economic factors, antenatal care and maternal characteristics on birth weight, prematurity, fetal death, date and cause of death in the five year of life, and growth in the first 5 years of life.	Measurement the effect of social economic factors on birth outcomes and child development nationally and over specific populations, according to age of mother, age of child, parity, geography, level of income, reception of Bolsa Familia, etc including:<br>Preparation of plan of analysis identifying all specific research questions of interest and detailing the methods to be used.<br>Perform analysis of data, interpretation and write up results and submission of the first 2 papers in high impact journals<br>Dissemination of findings with policy implications to policy makers	18-40 months<br>18-20 months<br>20-40 months<br>.<br>36-40 months.	US$<br>118,029.51
**Objective 3**<br>To estimate the impact of Bolsa Familia, on birth weight, prematurity, fetal death, mortality by age and cause and on growth on children in the first five years of	Measurement the impact of receiving BF, the value and the duration, on birth weight, prematurity, fetal death , mortality by age and cause and growth in the first years of life overall nationally and over specific	24-45 months	US$<br>111.302,30

life, overall and in sub populations and periods.	populations, including:<br><br>Preparation of plan of analysis identifying all specific research questions to be analyzed and detailing the methods to be used<br><br>Perform analysis of data, interpretation and write up results and submission of the first 2 papers in high impact journals<br><br>Dissemination of findings with policy implications to policy makers	24-26 months<br><br>26-45 months<br><br>42-45 months	
**Objective 4:**<br><br>To set up the strategic plan for the data center for the future including the financial sustainability and the plans for developing a public interface for the datacenter as a resource by researchers and decision makers	A clear strategic vision of the future of the cohort and the datacenter, of the public interface and mechanisms to assure long term future, including:<br><br>A clearly defined plan for the continuation and expansion of the cohort and data center<br><br>A detailed plan for the implementation of the public interface<br><br>A clearly defined strategic plan for long term funding and sustainability	36-48 months<br><br>36 -48 months<br><br>36-48 months<br><br>36-48 months	US$<br><br>78,788,20

Project Framework Narrative

__Overall Objective__ To conduct research on social effect and impact of social policies on healthy birth, growth and survival overall and in subgroups of interest in an unprecedented virtual cohort of 100 million Brazilians originated from CADU subjects included from 2007, in a properly established datacenter organized around safely linkage of CADU data with Bolsa Familia monthly payments, SINASC, SIM and growth monitoring from BF conditionalities, and to plan the development of aa sustainable public interface managing access to researchers and policy makers to this depository, offering methodological development, training and capacity building resources. Objectives 1 to 4 define the steps to achieve this.

OBJECTIVE 1:

Setup of the 100 million Brazilians cohort and data center

Description & assumptions

Data - The core of the Data Center will be a cohort of 100 million people who were interviewed between 2007 and 2012 for concession of social benefits (digitally recorded in the Cadastro Unico database, CADU) linked to 4 databases relevant to the assessment of healthy birth, growth and survival: Bolsa Familia monthly payments, SINASC, with records of all births, SIM, information on all deaths, and growth monitoring from BF conditionalities (with growth measurements measured as part of control of BF). Ethical approval must be obtained and datasets acquired.

Data Center - Setting up a data center of this magnitude requires careful preparation. The space needs to be carefully designed according to rules to give full physical protection for the data as well as to manage access to the relevant personnel. The computing capacity for data with identifies and for anonymized dataset, as well for providing access needs to be carefully defined. The software must be appropriate for the magnitude of the data base and for the functions of the center, ranging from receiving the data, managing it, cleaning, linking, updating, maintenance, and provision of anonymized datasets and carefully documenting

meta-data and recording access to the dataset. All activities must be carefully planned and codified in standard operation procedures.

Cohort - The data must be reorganized as a cohort with data about each specific individual at baseline and over the follow-up period.

Assumptions: The data exist, are of good quality, are transferable given the legal and regulatory environment. Ethical approval is routinely given for identifiable data when good procedures and assurances are in place. Given the group's established reputation for responsible and rigorous research, we assume that this will be completed soon.

Activities

To obtain full ethical approval and to acquire remaining data sets. We will apply for ethical approval. An agreement was signed between the Ministry of Social Development, FIOCRUZ President and Rectors of UFBa and UNB to give us copy of CADU and BF payments; GM will be obtained as an extension of this agreement. We are in advanced negotiations with the Ministry of Health in particular the Department for health information (DATASUS) for access to the remaining datasets, SINASC and SIM after ethical approval.

To setup the datacenter, to obtain relevant hardware and software, to develop probabilistic linkage algorithms and to define standard operation procedures (SOP). We plan to use a specially designated area in FIOCRUZ, sufficient to house secure facilities for the server handing non-anonymized datasets. We will acquire a computing cluster, with sufficient processing and storage capacity and adequate software; we will develop algorithms to perform data linkage with efficiency and accuracy, to produce anonymized datasets for analysis. The standard operation procedures (SOPs) for data manipulations, for data cleaning, for linkage, for keeping track of changes, and for producing meta-data for all the databases will be defined. Pilots are ongoing with one year of CADU dataset.

To define the 100 million Brazilians Cohort and link the cohort to births, deaths and growth databases. This dynamic cohort consists of all families registered in the CADU at any time in the period 2007 to 2012 and includes over 100 million individuals. Linking with SINAC will bring all the births from the members of the cohort; linkage with SIM will bring all those who died in their first five years of life, including still births For those children born from `Bolsa Familia` recipient families, repeated measurements of weight and height for the first 5 years of life as regular growth monitoring is one of the conditionalities of the program. SOPs will be produced to define the cohort structure. A profile will be written up describing the baseline and follow up characteristics of the cohort. Data will be done respecting rigorous safety procedures

Results and critical milestones

Results: A functioning datacenter with the cohort ready for analysis

Milestone 1a Ethical clearance granted and the five datasets (CADU, BF payments, SINASC, SIM, and GM) in our possession.

Milestone 1b Functioning datacenter, with adequate space, necessary computing facilities, tested software, final SOPs for linkage, data cleaning and validation and data extraction

Milestone 1c Cohort ready for data analysis. Paper on the cohort profile submitted; .

Results and measurements

Milestone 1a Ethical clearance granted and the five datasets (CADU, BF payments, SINASC, SIM, and GM) in our possession and 0- 6 months.

Milestone will be considered achieved if we have ethical approval for this analysis and we obtained the 5 datasets in full.

Milestone 1b Functioning datacenter, with adequate space, necessary computing facilities, tested software, final SOPs for linkage, data cleaning and validation and data extraction. 0-12 months

Milestone will be considered achieved if we have a datacenter with adequate space and facilities, software and SOPs.

Milestone 1c Cohort ready for data analysis. Paper on the cohort profile submitted; 6-18 months

Milestone will be considered achieved if the cohort is ready for analysis (baseline data from CADU linked to BF monthly payment, SINASC, SIM and Growth, and a proof of concept analysis conducted on one outcome) and a paper describing the cohort profile is submitted for publication

OBJECTIVE 2:

To estimate the effect of social and economic factors, antenatal care and maternal characteristics on birth weight, prematurity, fetal death, date and cause of death in the five year of life, and growth in the first 5 years of life.

Description
Research on the effect of social economic characteristics and antenatal care: To estimate the effect of the large range of socio economic variables contained in CADU (which are collected to assess if the family qualifies for receipt of social benefits) and of maternal characteristics contained in SINASC (ante natal care, parity and schooling) on the child's birth weight and prematurity, fetal death, date and cause of death in the first year of life, and for recipients of Bolsa Familia, effect on growth in the first 5 years of life. This would be done nationwide initially but given the amount of information this could be repeated separately for specific populations, according to age of mother, age of child, parity, geography, level of income, reception of bolsa familia, etc

Assumptions Data is available and of good quality**.** Size of the cohort is unprecendently large, and it is sufficient not only for overall analysis but also for fine stratification. Cohort was successfully constructed. Statistical methods are well established and developed to data of this magnitude.

Activities
Analysis of the cohort will use statistical methods for longitudinal data analysis, such as estimation of rates according to exposure, Cox regression, Poisson regression, GEE and mixed methods, as well as structural equation modeling, for instance, to investigate the causal pathways (27,28). These methodologies can take into account person time at risk, duration and level of exposure, time lag from exposure to effect, intergeneration effects and family effects. The size of the cohort will allow analysis of the role of social and health care variables separately in fine subgroups and different time periods on each of the outcomes studied: prematurity, low birth weight, fetal death, growth pattern and mortality overall and for relevant causes.

Results Measurement the effect of social economic factors on birth outcomes and child development nationally and over specific populations, according to age of mother, age of child, parity, geography, level of income, reception of bolsa familia, etc
Critical Milestones:

Milestone 2a- Preparation of plan of analysis identifying all specific research questions to be analyzed and detailing the methods to be used.

Milestone 2b- Perform analysis of data, interpretation and write up results

Milestone 2c Submission of the first 2 papers in high impact journals

Milestone 2d- Dissemination of findings with policy implications to policy makers

Results Measurement

Milestone 2a- Preparation of plan of analysis identifying all specific research questions of interest and detailing the methods to be used. 18-20 months.

Milestone will be considered achieved if the plan is produced and approved by the scientific committee.

Milestone 2b- Perform analysis of data, interpretation and write up results and submission of the first 2 papers in high impact journals 20-40 months

Milestone will be considered achieved if at least two papers were submitted and are under review or further on the editorial process.

Milestone 2c- Dissemination of findings with policy implications to policy makers 36-40

Milestone will be considered achieved if we conducted at least two presentations to high officers in Brazilian governments or International organizations

OBJECTIVE 3:

To estimate the impact of Bolsa Familia on birth weight, prematurity, fetal death, mortality by age and cause, and on growth on children in the first five years of life, overall and in subpopulations and periods.

Description
To estimate the impact of receiving Bolsa Familia, a cash transfer program, on child's birth weight and prematurity, fetal death, growth and mortality by age and cause of death in the first five years of life. Child growth analysis will be restricted to children from BF recipients' families since data is collected as part of the systematic monitoring of compliance with BF conditionalities. This would be done nationwide initially but given the size of the cohort could be repeated separately for specific populations, according to age of mother, age of child, parity, geography, level of income,etc

Assumptions
Data is available and of good quality, including data on the social intervention - BF. Size of the cohort is unprecedently large and sufficient not only for overall analysis but also for fine stratification. Cohort was successfully constructed. Statistical methods are well established. The development of refined methodological alternatives for evaluation of interventions can be adapted to data of this magnitude.

Activities
The analysis will investigate the impact of receiving or not BF, and different levels of support and duration of receiving the benefit, on prematurity, birthweight, fetal death, mortality in the first five years of life overall and by cause, and growth (restricted to those in receipt of BF) .

The classical approach to study impact of new interventions is the trial. In Brazil, as in many other countries, randomized controlled trials for poverty-reduction interventions are not feasible - this is particularly true in Brazil, where the program is one of the largest and most successfully implemented cash transfer programs in the world. The data exists and it is of an unprecedented richness and volume. There is a long tradition of using quasi-experimental designs for evaluation of impact in health and economic fields (19,20,21,22). The analysis proposed will include all beneficiaries and all those who were screened for benefits, with documented heterogeneities of conditions. The proposed methodologies will provide a robust control of any selection biases. CADU record of the family and individuals at entry– which contains a wide range of socioeconomic characteristics of the subjects before receiving (or not) the BFP benefits - will represent the cohort baseline. Two analytical approaches will be used: regression discontinuity design (RDD) and propensity score matching (PSM). The regression discontinuity design (RDD) is an econometric quasi-experimental design that have strong connections with other causal inference estimation procedures in statistical research and it is considered one of the most accurate methodologies for impact evaluation using observational data. The RDD can be applied in any context where a particular intervention is administered according to a pre-specified rule linked to a continuous variable referred to as the assignment variable, in this case, Bolsa Familia. This is only possible because the introduction of the BF can be treated as a quasi-experimental setting, where recipients (and non recipients) who are close to the eligibility cut off point can be treated as subject to a `quasi randomization `. The impact on subjects outside the RDD selected bandwidth will be evaluated using Propensity Score Matching. The longitudinal dimension of the big dataset, with approximately 100 million subjects – 50% of them BFP beneficiaries at some time during the period- from 2007 to 2012, will be explored using a difference-in-difference design(DD)-RDD and DD-PSM approaches at different points in time and in different subgroups (29,30,31,32).

Results Measurement the impact of receiving BF, the value and the duration, on on birth weight, prematurity, fetal death , mortality by age and cause and growth in the first years of life overall nationally and over specific populations

Critical Milestones:

Milestone 3a- Preparation of plan of analysis identifying all specific research questions to be analyzed and detailing the methods to be used.

Milestone 3b- Submission of the first 2 papers in high impact journals

Milestone 3c- Dissemination of findings with policy implications to policy makers

Results Measurement

Milestone 3a- Preparation of plan of analysis identifying all specific research questions to be analyzed and detailing the methods to be used. 24-26 months

Milestone will be considered achieved if the plan is produced and approved by the scientific committee.

Milestone 3b- Perform analysis of data, interpretation and write up results and submission of the first 2 papers in high impact journals 26-45 months

Milestone will be considered achieved if at least two papers were submitted and are under review or further on the editorial process.

Milestone 3c- Dissemination of findings with policy implications to policy makers 42-45 months

Milestone will be considered achieved if we conducted at least two presentations to high officers in Brazilian governments or International organizations

OBJECTIVE 4 :

To set up the strategic plan for the data center for the future including the financial sustenability and the plans for developing a public interface for the datacenter as a resource by researchers and decision makers.

Description

Future of the cohort and datacenter - Once the cohort and datacenter is established for research on healthy birth, child growth and survival, it will be extremely cost effective to expand it to include subsequent years of follow-up, other age groups (expansion to late childhood, adolescence and adulthood), other health sector data (over five mortality overall and by cause, hospitalizations, primary health care, vaccinations, cancer registration, infectious diseases notifications, etc.) followed by data from other domains: education (eg school attendance and performance), housing, employment, marriage and divorce, crime etc.

Future as a public interface - We aim to set up a public interface to the data center to support research from other scientists focused on different aspects of impact of social interventions and other social determinants on health and other outcomes. This requires careful planning of the many aspects involved: setting up the facilities for providing anonymized datasets for researchers and policy makers, setting up a process for consideration (ethical and scientific) of each request, definition of rules for access to the anonymized data, planning for the necessary staff to keep the process functioning. We also envisage the datacenter to function as a center for methodological development , training and capacity building.

A sustainable future - We aim at the end of this funding period to have established a public profile and develop a clear vision of financial sustainability and governance that assure a long term perspective for the cohort and the data center.

Assumptions: We assumed that the first three objectives were met; there is great interest on the 100 million Brazilian cohort from the national and international scientific and policy makers communities given its demonstrated potential.

Activities:
To set up the strategic plan for the future, including: a) detailed plans for the continuation and expansion of the cohort and data center; b) detailed plans for building up the public interface including aspects of computing, governance (scientific and ethics) and financial implications; c) detailed plans for sustainability and long term funding; d) to promote short courses, seminars, visits, postgraduate training (MSc and PhD) stimulating scientific and methodological development around the study of impact of social policies and other social determinants.

Results

To have a clear strategic vision of the future of the cohort and the datacenter, of the public interface and mechanisms to assure long term future and have developed plans to achieve this.

Critical Milestones

Millestone 4a - : A clearly defined plan for the continuation and expansion of the cohort and data center

Millestone 4b - A detailed plan for the implementation of the public interface

Millestone 4c - A clearly defined strategic plan for long term funding and sustainability

Results Measurement

Millestone 4a - : A clearly defined plan for the continuation and expansion of the cohort and data center 36-48 months

Millestone 4b - A detailed plan for the implementation of the public interface 36 -48 months

Millestone 4c - A clearly defined strategic plan for long term funding and sustainability 36-48 months

Milestones 4a,4b and 4c will be considered achieved if the plans are produced and approved by the scientific and advisory committees

Challenges for the four objectives: a) There is at international level discussions on the legal regulatory framework for the access to non-anonymized data. The strategy will be for the group is to keep updated in this debate and contribute to it in Brazil and internationally. b) The analysis of quasi-experimental and evaluative studies is a fast moving field The strategy will be for the group to be an active partner in this methodological and conceptual development. c) The societal expectation on ethics and governance for public access to anonymized data is changing quickly The strategy will be for the group to provide a plan for the public interface that is forward looking.

V. Organizational Capacity and Management Capability

Provide a brief description of:

1. The organization's history, mission, and comparative advantage to implement this project. (If a consortium is submitting the proposal, please provide this information <u>for each organization</u> in the consortium.)
Fundação Oswaldo Cruz - FIOCRUZ is a leading institution in health research in Brazil, linked with the Brazilian Ministry of Health. FIOCRUZ´s main campus is in Rio de Janeiro and there are campi in several Brazilian State's capitals, including Salvador. FIOCRUZ is composed of several research institutes covering research on biomedical, clinical, public health and social sciences health research and production of vaccines and drugs. The PI has large experience in design and conduct complex epidemiological and evaluative studies with projects supported by national and international organizations with over 350 peer reviewed publications. Part of his research has been devoted on aspects of child growth and development. He is the senior author of a seminal paper on the impact of Bolsa Familia Program on child mortality and hospitalization. He has links with the Instituto de Saude Coletiva - Federal University of Bahia, where he is a Permanent Professor of the Postgraduate Program in Collective Health at UFBa and is a Senior Researcher of FIOCRUZ,. His research activities are based in both FIOCRUZ and ISC-UFBa. In FIOCRUZ he is based at the Gonçalo Muniz Research Center in Salvador-Bahia and is associated with the Center for Information

and Policies on Social Determinants of Health at the National School of Public Health - FIOCRUZ, Rio de Janeiro. Are also from FIOCRUZ Carlos Teles (a Statistician with great experience in analysis of large epidemiological studies, will be involved in data analysis and interpretation), Yury Ichihara (Epidemiologist) and Gerson Penna (Dr. Gerson Penna is a senior public health expert with great experience in large routine dataset management and study of poverty related diseases.)

Instituto de Saúde Coletiva - ISC-UFBA ISC is an important center of Public Health research based in Salvador with a highly recognized PostGraduated Program in Public Health evaluated in the top rank (seven) by the Brazilian CAPES. Also on UFBA, are Dr. Marco Barreto, Associated Professor in Computing will lead on linkage and data security. Dr. Darci Santos is a research on child development will help in data analysis and interpretation. Other collaborators from UFBa include Drs Rosana Aquino (epidemiologist), Davide Rasella (epidemiologist) Rosemeire Fiaccone, e Leila Denise Amorim (statistician with great deal of experience of analysis of big and complex data sets, will be involved in data management, analysis and interpretation).

LSHTM and the Farr Institute: The London School of Hygiene and Tropical Medicine is one of the leading schools of public health, with a long established record of research on inequalities, evaluative research, on research using linked routine data - including Big Data and is a leader in development of new statistical methods. LSHTM mission is to contribute to the improvement of public health and health equity through rigorous research teaching and influencing policy. The main LSHTM PI is Laura Rodrigues, a leading Professor of Epidemiology with substantive experience on large and complex epidemiological and evaluative studies, including neonatal epidemiology, and in the use of routine data. Laura had a long-term collaboration with Mauricio Barreto and other groups in Brazil. She is a Special Visiting Researcher at UFBa and that is associated with her presence in Brazil frequently, with expected 10 to 25% of her time in Brazil. Her and LSHTM expertise will be of great relevance to the success of the program and to reach several of the milestones. She will contribute directly to the work and will provide a link to the exciting collaborative research environment at the LSHTM, with , a critical mass of epidemiologists, statisticians and social scientists, and a tradition of multi disciplinarily reflected in 8 cross faculty centers; of particular relevance here are the Center for Methodology, lead by Prof Bianca de Stavola, the Center for Evaluation, lead by Dr James Hargreaves, and MARCH, The Center for Maternal, Adolescent, Reproductive, and Child Health (MARCH) led by Prof Joy Lawn, who also leads Saving Newborn Lives and is a collaborator in this project. LSHTM has a long history of international collaboration, and specifically with Brazil. LSHTM co- host The Farr Institute of Health Informatics Research which aims to deliver high-quality, cutting-edge research linking electronic health data with other forms of research and routinely collected data, as well as build capacity in health informatics research. Prof Liam Smeeth, the co-director of the London Farr Institute is a professor of Epidemiology at the LSHTM and a collaborator in the project.

Additional institutions and co-Pis include: Dr. Romulo Paes de Souza an epidemiologist with great experience in inequalities studies and a world expert in cash-transfer and other social programs, based at the World Center for Sustainable Development (Rio+ Center)/UNDP, Rio de Janeiro-Brazil, and Professor Marcia Furquim de Almeida from School of Public Health, University of Sao Paulo, an expert in neonatal health, and a member of Technical Advisory Committee of Mortality and Live Birth Information System; and a member of the Evaluation Committee of Health Surveillance Department, both in the Brazilian Ministry of Health. She will contribute her expertise to the data management, analysis and interpretation, particularly on birth outcomes and neonatal health .

2- Previous experience in developing, implementing, and managing projects in the geographical and technical areas proposed. (If a consortium is submitting the proposal, please provide this information <u>for each organization</u> in the consortium.)

The team has world recognized ability to manage this project. All the main institutions involved are large research and training centers with leadership in several different public health and epidemiology research areas and involved in large isolated or collaborative projects.

Prof Mauricio Barreto led several large epidemiological and evaluative projects. It includes: a) a randomized control trial for evaluating the effect of Vitamin A supplementation on children diarrhea and pneumonia (supported by INAN-MS, UNICEF and WHO); b)evaluation of the impact of a sanitation program (Baia Azul) in the City of Salvador-Bahia (supported by Bahia State Government, CNPq); c) a National Institute of

Science and technology centered in innovation in health. Prof Laura Rodrigues led complex and large studies in the UK and internationally, including one of the first studies of maternal to child transmission of HIV in Brazil, funded by the Overseas Development Agency, UK; the `IID study, epidemiology`, a national study of infections intestinal disease in the community, in primary health care and notifications in the UK, funded by the Foods Standard Agency, a project on verbal autopsy for adult mortality in Africa , funded by the World Bank, and a study of duration of BCG protection in children vaccinated at birth and at school age, in the UK, funded by the National Institute for Health Research. Laura Rodrigues and Mauricio Barreto also have a record of collaborations, having managed jointly 2 very large and complex research programs in Brazil: A randomized trial with over 300 thousands participants to evaluate the effect of BCG revaccination at school age (REVAC-BCG) (supported by DFID-UK and MoH-Brazil), which provided information that led to a change in the policy of BCG revaccination in Brazil; and the Wellcome Trust Program "Social Change, Allergy and Asthma in Latin America(SCAALA)", based on epidemiological studies in Brazil and Ecuador children, including an infant cohort with resulted in over 50 publications so far.

The 100 million cohort and datacenter project will be based analysis of a large cohort resulting from linkage of different routine electronic datasets. The team has expertise in IT, led by Marcos Barreto Professor of Information Technology in Universidade Federal da Bahia and with a team of young bright computer scientists; Marcia Furquim de Almeida has extensive research using linked birth and mortality data, and is member of the advisory committee of the government unit responsible for producing routine mortality and birth data in Brazil. Liam Smeeth is the vice director of the FARR Institute, funded by the UK Government and Research Councils to deliver high-quality, cutting-edge research linking electronic health data with other forms of research and routinely collected data, as well as build capacity in health informatics research.

The research proposed here is the evaluation of effect of social inequalities and the impact of social policies in healthy birth and child nutritional status and development. All together the research team accumulate experience to cover all the challenges involved in this field of research. The PIs (Mauricio Barreto and Laura Rodrigues,) and other collaborators (Romulo Souza, Liam Smeeth, Gerson Penna) has a solid background on evaluative research and on inequalities and health. The majors institutions involved (FIOCRUZ, ISC and LSHTM) has long tradition on evaluative and health inequalities research; Joy Lawn in an internationally recognized leader of research on perinatal mortality, and Marcia Furquim, Laura Rodrigues, have published extensively on perinatal and neonatal epidemiology in Brazil and internationally. Mauricio Barreto and Darci Neves have undertaken research on cognitive development. Rita Silva, Ana Duran, Mauricio Barreto, Laura Rodrigues and others have a great deal of experience on studies related to nutritional status and childhood diseases in childhood.

3-If there is a parent or administering institution to which the team undertaking this project will report, please describe any specific support required by the institution for implementation of the project and confirm that it will be provided.
The lead institution is Centro de Pesquisa Gonçalo Muniz- FIOCRUZ in Salvador; their grants are administered by FIOTEC, witch requires a 10% indirect costs. There are partners institutions but for this project they are willing to contribute their effort without requiring funds, so there will be no subcontracts.
.
4. **Describe the facilities, resources and equipment available for the proposed project at all participating sites. Provide enough detail to guarantee the infrastructure is sufficient to allow the project to be carried out including the accounting systems and ability to make and supervise sub-grants and contracts. (If a consortium is submitting the proposal, please provide this information for each organization in the consortium.**)

The main basis of the project will be the Centro de Pesquisa Gonçalo Muniz- FIOCRUZ in Salvador; it has space for the computing center and office for researchers and technicians. The computing center has a server with small storage capacity. Resources from the project will fund the expansion of existing computing resources. Complementary activities will be based in the Instituto de Saude Coletiva and Dept. of Statistics and Computing of the Federal University of Bahia. Between them, these institutions have the resources and facilities to house the project, new equipments and personnel. The Funds of the project will be administered by FIOTEC a foundation responsible for administering research funding contracted with all FIOCRUZ units. FIOTEC is a well established and reputable organization, with more than 15 years of experience; FiOTEC administers around 100 million dollars of grant funding every year.

B. Management and Staffing Plan for this Project

Management structure

The project will be led by the PI, Mauricio Barreto and a small executive committee, consisting of the LSHTM PI, Laura Rodrigues, and the coordinators of three working groups (IT, Research and Cohort Administration), with all staff responding to one of the coordinators. This will be supported by a Scientific Committee which includes all co-PIs and an Advisory Committee to be appointed, but which will includes the two collaborators, Joy Lawn, from LSHTM (MARCH) and from Saving Newborn Lives and March, and Liam Smeeth from the LSHTM and the Farr Institute.

Capability of the management team to manage the project: Prof Mauricio Barreto and Prof Laura Rodrigues have demonstrated ability to manage large projects, and together or separately led sucessfully many large research programs, including the BCG REVAC trial, , with 300 000 subjects, a Wellcome Trust program SCAALA , (Social Change, Allergy and Asthma in Latin America) , with over 50 publications, etc Yury Ichihara has excellent management skills and has recently completed a large multicenter study of evaluation of rotavirus vaccine and will coordinate the research component of the project . Prof Marcos Barreto is a Professor of Information Technology in the Computing Department at UFBA and will coordinate the IT component of the project. We plan to hire an executive officer to administer the cohort.

Overview of decision making plans and processes.
Day to day decision will be taken by the staff under coordination of the area coordination. The executive committee will meet monthly in person or skype to review progress and set up the plans for the following period, within the strategic plan for the duration of the project. the scientific committee will meet every 6 months, and the advisory committee will meet annually, to receive a report of activities progress, review strategic priorities and plans.

Organizational chart

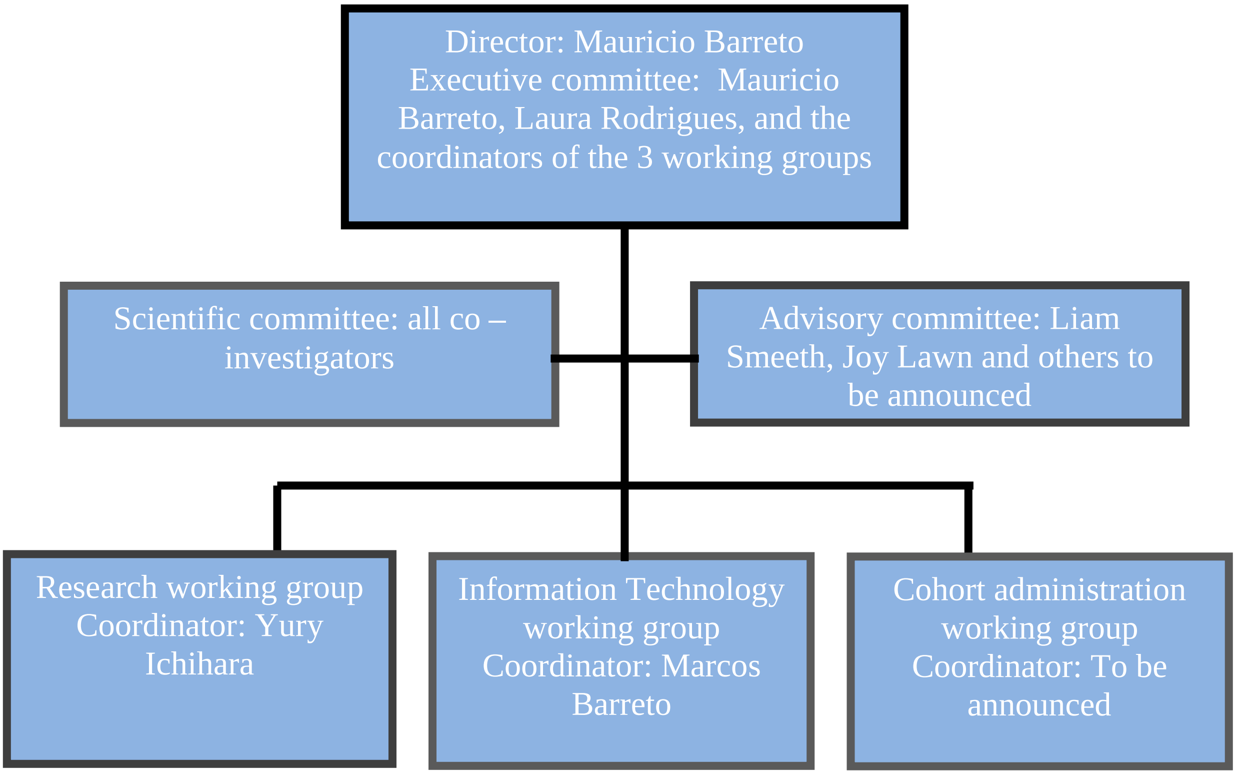

Participating institutions
The research will be conducted at FIOCRUZ with joint leadership from London School of Hygiene and Tropical Medicine/ LSHTM, others participating institutions include the Universidade Federal da Bahia, the

World Center for Sustainable Development (Rio+ Center)/UNDP, with collaborators from University of São Paulo

Approach to furthering global initiative objectives

a) Active dissemination of findings to policymakers and scientists
Dissemination of findings will be done actively, using not only scientific publications in international and national high impact journals but also through meetings with national and international policymakers. Given the interest of the Ministry of Social Development in the 100 million Cohort , we expect full government facilitation for the dissemination of our findings. We aim to develop a strong digital presence with a website, and active use of electronic social media and other media .

b) Datacenter to serve as beacon for scientific interchange, training and capacity strengthening and for methodological development.
The datacenter will play an important role in leading methodological developments as well as a center for training and capacity building, with short courses, symposia and workshops; it will also receive , MSc and PhD students interested in undertaking their projects on the cohort. .

c) Development of the public interface to enable access to anonymized data
In the last year of the project we aim develop the plans for setting up a public interface to the data center to provide access to anonymized data sets to support research from other scientists focused on different aspects of impact of social interventions and other social determinants on health and other outcomes. This requires careful planning of the many aspects involved: setting up the facilities for providing anonymized datasets for researchers and policy makers, setting up a process for consideration (ethical and scientific) of each request, definition of rules for access to the anonymized data, planning for the necessary staff to keep the process functioning.

Managing the technology to **further the global initiative objectives**
There is no IP associated with the project. One of the products of this project will be the production the 100 million cohort and datacenter, an unprecedented resource for research on social determinants of health and evaluation of impact of social intervention on health. One of the objectives of the project is to plan for the development of the public interface for the cohort and datacenter. Given our experience after two years, and the skills and knowledge of the executive, scientific and advisory committee, we have resources to develop the plan (in terms of IT, planning for required resources, definition of governance and ethics processes)

Citations, Appendices and Modules do not count against the 20-page limit.

VI. Citations

1- Commission on Social Determinants of Health. Closing the Gap in a Generation: Health Equity through Action on the Social Determinants of Health. Final Report of the Com- mission on Social Determinants of Health. Geneva, Switzer- land: World Health Organization; 2008.
2- Victora CG, Wagstaff A, Schellenberg JA, Gwatkin D, Claeson M, Habicht JP. Applying an equity lens to child health and mortality: more of the same is not enough. Lancet. 2003;362(9379):233-41.
3- Marmot M. Fair Society, Healthy lives: Published by "The Marmot review". London 2010
4- Paim J, Travassos C, Almeida C, Bahia L, Macinko J. The Brazilian health system: history, advances, and challenges. Lancet. 2011 May 21;377(9779):1778-97.
5- Victora CG, Aquino EM, do Carmo Leal M, Monteiro CA, Barros FC, Szwarcwald CL. Maternal and child health in Brazil: progress and challenges. Lancet. 2011 May 28;377(9780):1863-76.
6- Barreto ML, Rasella D, Machado DB, Aquino R, Lima D, Garcia LP, Boing AC, Santos J, Escalante J, Aquino EM, Travassos C. Monitoring and evaluating progress towards Universal Health Coverage in Brazil. PLoS Med. 2014 Sep 22;11(9):e1001692.5
7- Aquino R, de Oliveira NF, Barreto ML. Impact of the family health program on infant mortality in Brazilian municipalities. Am J Public Health. 2009 Jan;99(1):87-93.
8- Rasella D, Aquino R, Barreto ML. Reducing childhood mortality from diarrhea and lower respiratory tract infections in Brazil. Pediatrics. 2010 Sep;126(3):e534-40.
9- Rasella D, Aquino R, Santos CA, Paes-Sousa R, Barreto ML. Effect of a conditional cash transfer program on childhood mortality: a nationwide analysis of Brazilian municipalities. The Lancet 2013; **382**: 57–64.

10- Shei A. Brazil's Conditional Cash Transfer Program Associated With Declines In Infant Mortality Rates. Health Aff (Millwood) 2013; **32**: 1274–81.
11- Lagarde M, Haines A, Palmer N. Conditional cash transfers for improving uptake of health interventions in low- and middle-income countries: a systematic review. JAMA. 2007 Oct 24;298(16):1900-10.
12- Robertson L(1), Mushati P, Eaton JW, Dumba L, Mavise G, Makoni J, Schumacher C, Crea T, Monasch R, Sherr L, Garnett GP, Nyamukapa C, Gregson S. Effects of unconditional and conditional cash transfers on child health and development in Zimbabwe: a cluster-randomised trial. Lancet. 2013 Apr 13;381(9874):1283-92.
13- Campello T, Neri MC. Programa Bolsa Família: uma década de inclusão e cidadania IPEA, Brasilia 2013.
14- Ranganathan M, Lagarde M. Promoting healthy behaviours and improving health outcomes in low and middle income countries: a review of the impact of conditional cash transfer programs. *Prev Med* 2012; **55** (suppl)**:** S95–105.
15- Barham T. A healthier start: The effect of conditional cash transfers on neonatal and infant mortality in rural Mexico. *J Development Econ* 2011; **94:** 74–85
16- Paes-Sousa R, Santos LMP, Miazaki ÉS. Effects of a conditional cash transfer program on child nutrition in Brazil. Bull World Health Organ 2011; 89:496–503.
17- Duarte GB, Sampaio B, Sampaio Y. Programa Bolsa Família: impacto das transferências sobre os gastos com alimentos em famílias rurais. RESR. 2009; 47(4):903-918.
18- Nery JS, Pereira SM, Rasella D, Penna ML, Aquino R, Rodrigues LC, Barreto ML, Penna GO. Effect of the Brazilian conditional cash transfer and primary health care programs on the new case detection rate of leprosy. PLoS Negl Trop Dis. 2014 Nov 20;8(11):e3357.
19- Gertler PJ, Premand P, Martinez S, Rawlings LB, Vermeersch CMJ. Impact evaluation in practice. , The World Bank, 2010.
20- Khandker, SR. Handbook on impact evaluation : quantitative methods and practices. World Bank, Washington 2010
21- Shadish, WR, Cook, TD, Campbell DT. Experimental and Quasi-Experimental Designs for Generalized Causal Inference. Houghton, Boston 2002
22- Rockers PC, Røttingen JA, Shemilt I, Tugwell P, Bärnighausen T. Inclusion of quasi-experimental studies in systematic reviews of health systems research. Health Policy. 2015 Apr;119(4):511-21.
23- http://www.mds.gov.br/biblioteca/secretaria-nacional-de-renda-de-cidadania-senarc/cartilhas/cartilha-do-cadastro-unico/arquivo/Cartilha_CADASTRO_UNICO.PDF/download. Assessed 18.05.2015
24- Science Europe. How to Transform Big Data into Better Health: Envisioning a Health Big Data Ecosystem for Advancing Biomedical Research and Improving Health Outcomes in Europe. A workshop report, Ericie, Italy 2014
25- Szreter S. The Importance of Social Intervention in Britain's Mortality decline c.1850-1914: a Re-interpretation of the Role of Public Health. Soc Hist of Med, 1:1-38, 1988
26- Szreter S. Rethinking McKeown: the relationship between public health and social change. Am J Public Health. 2002 May;92(5):722-5.
27- Twisk JR Applied longitudinal data analysis for epidemiology: a practical guide Cambridge, Cambridge, 2013
28- Fitzmaurice G et al. Longitudinal data analysis. Chapman & Hall, Boca Raton, 2009
29- Caliendo M, Kopeinig S. Some Practical Guidance for the Implementation of Propensity Score Matching. Journal of Economic Surveys 2008; **22**: 31–72
30- Lee D, Lemieux T. "Regression Discontinuity Designs in Economics". Journal of Economic Literature 2010;48:281-355.
31- Holmes WM Using propensity score in quasi-experimental designs. Sage, Thousands Oak,2014
32- Imbens GW, Rubin, DB. Causal inference in statistics, and in the social and biomedical sciences. Cambridge, Cambridge, 2015

VII. Appendices

A. Budget Spreadsheet

Submitted as a separate EXCEL FILE

B. Budget Narrative

To organize the cohort of 100 million Brazilians, we propose to create and maintain a data center, to provide secure housing to databases with identifiers, as well as anonymised data that will be available to researchers. The cost for this is US$ 314754, and that includes supply and installation of a safe room and infrastructure to house the data center, and providing technical support during the warranty period. The main components are: 1) Project Design; 2) Adaptation of physical facilities; 3) Safe Room (for physical protection, certified fire and burglary, against gas and water, moisture diffusion barrier and attenuation of magnetic fields, fire resistance); 4) Technical floor (removable panels, wire fences beds and openings for cables); 5) Climate Control (flexible and efficient system consisting of high-performance modules and high heat factor and precision air conditioning, remote air condensers); 6) power system (dual distribution frames with micro processers and breakers, tailor-made and identified cables, circuits for passage from the generator and nobreaks); 7) System of early fire detection (active monitoring of aerosols in the air, connected with fire control, high sensitivity and statistical analysis software detectors); 8) Fire control (flue gas suppression system, automatic operation and optical detectors integrated into the monitoring system; 9) Logical Cabling (Cable data and logic to the safe room); 10) Supervision and alarm (monitoring systems and alarm transmission; 11) Data Center (area 16 m2); 10) Installation and equipment; 11) Technical Support (maintenance service with 24 x 7x 365 availability, covering a secure room and all subsystems and components of data center security infrastructure).

In the first year of the project, maintenance of the data center will be under that contract. However, for the remaining years (2nd to 4th year) provision of individual services will be contracted for maintenance of the Data Center structure (US$106229) and maintenance (electrical, cabling, keychain, sealing, hydraulic, plaster , dividing etc.) of physical facilities of the space that will accommodate researchers and users of data center (US$34426).

For the computer center, we will acquire a high-performance server and large storage capacity (US$234098), and redundant nobreaks (US$6557) and power generation (US$26229). For the maintenance and recovery of the equipment we will hire service of US$59016. The STATA software version 14 with shared network for 15 users will be purchased to enable the handling and analysis of large databases (US$7868)
The consumables (office supplies, media for backup and other items) will be purchased to provide the necessary logistical support to the establishment and operation of the data center, as well as linking activities, database preparation, analysis, interpretation and elaboration of a scientific paper

For the implementation and validations of data Linkage (definition of final SOP for linkage, validation and data extraction, linking the following banks (SINASC, SIM, Bolsa familia payments and growth monitoring) to CadÚnico and complete the preparation of CadÚnico data and the link anonymized data sets, and make anonymized data sets accessible to researchers) as well as for the operation and maintenance of the working data center, we budget for the implantation of 3 fellowships DTI-B (US$35409) and 2 fellowships DTI -C (US$8655) lasting 12 months. In addition, we will 16 system analysts (4 service benefits for 3 months / year of US$47213) on payment for services.

To prepare the database linked to CadÚnico and support specific aspects of the data analysis, we will pay for services rendered for 16 contracts (4 lots of 3months / year of US$47213), to process data, perform cleaning and consistency of the data, and perform statistical analyzes and reports.

In such a large database there is a great demand for administrative activities, and we predict needing 16

short term contracts in administrative support area (4 providing administrative support for 3 months / year of US$28327) to perform routine administrative requests like procurement of materials, support the organization of workshops and meetings of national and international staff, carry out the organization of the documentation related to the project in four years, carry out contacts, organize reports and other support activities necessary.

To undertake study of the effect of social and economic factors, prenatal care, maternal characteristics and the impact of PBF in birth weight, premature birth, fetal death, cause of death in five years of life and growth in the first 5 years life we will awards 13 fellowships DTI-B (US$118032 being 5 fellowshps in the 2nd year, 5 fellowships in 3rd year and 3 fellowships in the 4th year) and 8 fellowships DTI-C (US$25967- and 3 fellowships in the 2nd year, 3 fellowships at 3 years and 2 fellowships in the 4th year) lasting 12 months. The support the planning of the future strategic plan for the data center, including the financial sustainability and the development of a public interface for the Data Center as a resource for researchers and to support the dissemination of study findings, we will use three fellowships DTI-B (US$35409) and 2 fellowships DTI-C (US$8655).

One important aspect of this project is to reach out and disseminate to government agencies, authorities, universities and national and international scientific community. To support the Data Center organization, preparation Cohort 100 million Brazilians, development of analytical methods, discussion of results preliminary and prepare the strategic plan for Data Center In this way, it estimated the performance of 24 national meetings (US$22103 to 6 meetings / year), 6 international meetings (US$23543 to 1 in the 1st year ; 2 in the 2nd year; 2 at 3 years and 1 year 4), and 2 workshops (US$10152). In addition, a workshop is planned in the last year of the project for the presentation of the study results (US$5076).

Finally, budgetary and financial resources of the project will be managed by FIOTEC, which requires an administration fee of US$98071.
