# Supplementary material for: Conditional cash transfer program and child mortality: A cross-sectional analysis nested within the 100 Million Brazilian Cohort
Source: PLoS Med. 2021 Sep 28;18(9):e1003509. doi: 10.1371/journal.pmed.1003509 (PMC8478244; doi:10.1371/journal.pmed.1003509)
Supplement: S2 Text — (DOCX) [file pmed.1003509.s007.docx]

# **S2 Text. Data linkage Information**

*Linkage steps*

To identify beneficiaries and non-beneficiaries of BFP, the 100 Million Brazilian Cohort baseline and BFP payroll were linked deterministically using a unique identifier (i.e. NIS). The cohort baseline, live births and mortality records were linked through four individual-level identifiers (child’s name [or mother’s name if child’s name was absent], child’s date of birth, child’s sex and municipality of residence) in three steps (Figure S1) using the CIDACS-RL linkage tool ([https://gitHub.com/gcgbarbosa/cidacs-rl](https://github.com/gcgbarbosa/cidacs-rl)). In the first step, records were linked using information from the children (name, sex, date of birth and municipality). In the second step and third steps, entries that were not linked on the first step were then submitted using information from mothers (name, age or date of birth and municipality). The entire process is based on a similarity score ranging from 0-1 between all the pairwise comparisons; records with the highest scores are then considered to be true linked pairs.

# **[S1 Fig. Linkage process.** Flowchart of the linkage process between the Cidacs’ 100 Million Cohort baseline, mortality data (SIM - Brazilian Mortality Information System) and live birth records (SINASC - Brazilian Live Birth Information System)]

To assess the accuracy of the linkage procedures a manual verification is performed, based on 2000 randomly selected pairs from all possible paired records. This manual verification is used to classify pairs as true or false matches. Using several cut-off points for sensitivity and specificity, these manually classified pairs are then compared to their algorithm-based linkage status to determine the sensitivity and specificity at each potential cut-off point (Table A, S2 Fig and S3 Fig). The cut-off that corresponds to the best (optimal) sensitivity and specificity scores is then chosen for the entire linkage process.

[**S2 Fig.** ROC curve for the linkage between deaths of children with ages 1- 4 years (with name on the death certificate) and the Cidacs' 100 Million Cohort baseline]

[**S3 Fig.** ROC curve for the linkage between deaths of children under five (without name on the death certificate - using the name of the mother) and the Cidacs' 100 Million Cohort baseline]

*Quality issues and limitations*

Quality issues in Brazilian death registries have been addressed by two [(13,15)](https://paperpile.com/c/at80TD/5zVvE+KMHVf) out of the four existing studies on the effects of BFP on child mortality, and in our case, this issue was also present. A total of 80,520 mortality records (out of 858,610 total) were not submitted to linkage with the 100 Million Cohort, due to missingness in the child’s name and unavailability of alternative linkage attributes such as the child’s date of birth and/or mother’s date of birth. Neonatal deaths were the biggest parcel of this number, comprising 77.3% (*N*=62,248) of non-submitted records and also having the biggest within-group percentage of non-submitted records, corresponding to 12.9% of all neonatal deaths (*N*=62,248 out of 484,143) (Table B).

To account for this possible bias and as a form of sensitivity analysis, we calculated the percentage of nameless death records submitted to linkage in each municipality and broke down our estimates into eight different groups: municipalities in which all nameless death records were submitted to linkage, municipalities in which 90.1 to 99.9% of all nameless records were submitted to linkage, in which 80.1 to 90% of nameless records were submitted, 60.1 to 80%, 40.1 to 60%, 20.1 to 40%, 0.1 to 20% and municipalities in which all nameless death records were excluded from linkage (0% group) (Table C).

**Table A - Linkage accuracy estimates based on manual review of a sample of linked records (N= 2000)**

| **Subgroup** | **Specificity (%)** | **Sensitivity (%)** |
| --- | --- | --- |
| Deaths of children between 1 to 4 years  (with name on the death certificate) | 88 .00 | 80 .10 |
| Deaths of under five children  (no name on the death certificate - using the name of the mother) | 97 .50 | 96 .40 |

#

# **Table B - Distribution of under-five mortality across records submitted and non-submitted to linkage with the Cidacs’ 100 Million Cohort**

|  | **Non-submitted to linkage** | **Submitted to linkage** | **Total** |
| --- | --- | --- | --- |
| **Neonatal deaths** | 62248 | 421895 | 484143 |
| % within Neonatal deaths  (row percent) | 12 .86 | 87 .14 | 100 |
| % within non-submitted or submitted records (column percent) | 7 .31 | 54 .22 | 56 .39 |
| **Post-neonatal deaths** | 13054 | 230555 | 243609 |
| % within Post-neonatal deaths | 5 .36 | 94 .64 | 100 |
| % within non-submitted or submitted records (column percent) | 16 .21 | 29 .63 | 28 .37 |
| **1-4 years** | 5218 | 125640 | 130858 |
| % within 1-4 year old deaths | 3 .99 | 96 .01 | 100 |
| % within non-submitted or submitted records (column percent) | 6 .48 | 16 .15 | 15 .24 |
| **Total** | 80520 | 778090 | 858610 |
| % within all deaths | 9 .38 | 90 .62 | 100 |
| % within non-submitted or submitted records (column percent) | 100 | 100 | 100 |

# **Table C - Estimates of the association between Bolsa Família Program (BFP) and child mortality (1 to 4 years of age), by municipal subgroups of % of nameless records submitted to linkage**

|  | **% of nameless records submitted to linkage** | **Weighted Odds Ratio**  **(95% CI)** | **p-value** | **Robust Standard Error** | **N** |
| --- | --- | --- | --- | --- | --- |
| **Beneficiary status (BFP=1)** | 100% | 1.36  (1.01-1.83) | 0.039 | 0.206 | 163,999 |
|  | 90.1-99.9% | 1.28  (0.93-1.75) | 0.129 | 0.205 | 94,721 |
|  | 80.1-90% | 0.77  (0.68-0.88) | <0.001 | 0.502 | 825,919 |
|  | 60.1-80% | 0.80  (0.73-0.88) | <0.001 | 0.368 | 2,291,481 |
|  | 40.1-60% | 0.85  (0.73-0.98) | 0.035 | 0.667 | 613,654 |
|  | 20.1-40% | 0.94  (0.74-1.20) | 0.616 | 0.116 | 230,474 |
|  | 0.1-20% | 0.89  (0.78-1.03) | 0.110 | 0.629 | 877,177 |
|  | 0% | 0.89  (0.78-1.03) | 0.135 | 0.640 | 211,564 |
|  | | | | |  |
