# Supplementary material for: Conditional cash transfer program and child mortality: A cross-sectional analysis nested within the 100 Million Brazilian Cohort
Source: PLoS Med. 2021 Sep 28;18(9):e1003509. doi: 10.1371/journal.pmed.1003509 (PMC8478244; doi:10.1371/journal.pmed.1003509)
Supplement: S3 Text — Information on propensity score estimation, kernel matching, subgroup analysis, and robustness check. (DOCX) [file pmed.1003509.s008.docx]

**S3 Text. Kernel matching procedure and robustness check**

Table A shows the estimates from the logit model for the probability of receiving BFP (propensity scores) based on baseline characteristics of the child’s mother and living conditions. S4 Fig shows the distribution of estimated propensity scores given the covariates. Overlapping histograms are displayed for the beneficiaries and non-beneficiaries. While the two groups differ, as expected, in both groups the support of the estimated propensity scores is nearly the entire unit interval.

# [**S4 Fig. Propensity scores common support area.** Distribution of propensity scores across beneficiaries and non-beneficiaries]

Tables B to E shows the unadjusted estimates for the same subgroups displayed on Tables 3 to 6 in the main text.

To justify our hypothesis that the association between BFP and child mortality varies across subgroups, we performed a statistical test for interaction by including BFP*subgroup indicator terms in all our subgroup models. The general results, including the likelihood ratio (LR) test to evaluate the interaction term, can be found on Tables F to I.

As a form of robustness check, we conducted additional inverse probability treatment weighting (IPTW) analysis. The unadjusted and adjusted estimates for these regression models can be found on Table J and the results for the analysis within subgroups of maternal race/skin color, gestational age at birth groups, municipal quintiles of per capita income (MHDI-R) and Cadastro Unico’s decentralized management index (Municipal DMI) can be found on Tables K to M.

# **Table A - Logistic models to estimate the propensity scores for Bolsa Família Participation**

| **Variables** | **Odds Ratio**  **(95% CI)** | **p-value** | **Standard Error** |
| --- | --- | --- | --- |
| **Maternal education** |  |  |  |
| ≤ 3 years | Ref. |  | - |
| 4-7 years | 1.27  (1.26 - 1.27) | <0.001 | 0.0031 |
| ≥ 8 years | 1.19  (1.18 - 1.20) | <0.001 | 0.0047 |
| **Maternal race/skin color** |  |  |  |
| White | Ref. | <0.001 | - |
| Black | 1.26  (1.24 - 1.27) | <0.001 | 0.0071 |
| Asian descent | 1.04  (1.00 - 1.07) | <0.001 | 0.0179 |
| Mixed/brown | 1.14  (1.13 - 1.14) | <0.001 | 0.0029 |
| Indigenous | 4.63  (4.44 - 4.82) | <0.001 | 0.0999 |
| **Maternal age** |  |  |  |
| 20-34 | Ref. | <0.001 | - |
| ≤ 19 | 1.14  (1.14 - 1.15) | <0.001 | 0.0032 |
| ≥ 35 | 0.75*  (0.74 - 0.76) | <0.001 | 0.0035 |
| **Maternal marital status** |  |  |  |
| Has a partner (married, in a relationship) | Ref. | <0.001 | - |
| No partner (single, divorced, widowed) | 1.45  (1.44 - 1.46) | <0.001 | 0.0032 |
| **Maternal parity** |  |  |  |
| no child | Ref. | <0.001 | - |
| 1 child | 1.57  (1.56 - 1.58) | <0.001 | 0.0041 |
| 2 children | 2.45  (2.43 - 2.46) | <0.001 | 0.0086 |
| 3 children | 3.56  (3.53 - 3.60) | <0.001 | 0.0186 |
| 4 children or more | 5.15  (5.09 - 5.21) | <0.001 | 0.0309 |
| **Household density** |  |  |  |
| ≤ 2 people per room | Ref. | <0.001 | - |
| > 2 people per room | 1.88  (1.86 - 1.91) | <0.001 | 0.0141 |
| **Region** |  |  |  |
| North | Ref. | <0.001 | - |
| Northeast | 1.36  (1.35 - 1.37) | <0.001 | 0.0052 |
| Southeast | 0.90  (0.89 - 0.91) | <0.001 | 0.0034 |
| South | 0.59  (0.58 - 0.60) | <0.001 | 0.0028 |
| Center-West | 0.58  (0.57 - 0.58) | <0.001 | 0.0028 |
| **Registration year in Cadastro Unico** | 0.93  (0.93 - 0.94) | <0.001 | 0.0003 |
| **N** | 5,426,291 |  |  |

#

#

#

#

# **Table B - Regression results: Coefficients on unadjusted kernel weighted logistic regressions within subgroups¹ of municipal quintiles of per capita income (MHDI-R).**

| **MHDI-R** | **Weighted Odds Ratio***  **(95% CI)** | **Robust**  **Standard Error** | **p-value** | **N** |
| --- | --- | --- | --- | --- |
| **Model b1** |  |  |  |  |
| 1st quintile  (lowest income) | 0.71  (0.62 to 0.82) | 0.050 | < 0.001 | 714,779 |
| Constant | 0.004  (0.003 to 0.005) | 0.0004 | < 0.001 |  |
| **Model b2** |  |  |  |  |
| 2nd quintile | 0.75  (0.66 to 0.85) | 0.047 | < 0.001 | 803,236 |
| Constant | 0.003  (0.003 to 0.004) | 0.0003 | < 0.001 |  |
| **Model b3** |  |  |  |  |
| 3rd quintile | 0.84  (0.73 to 0.98) | 0.062 | 0.026 | 722,599 |
| Constant | 0.002  (0.002 to 0.003) | 0.0002 | < 0.001 |  |
| **Model b4** |  |  |  |  |
| 4th quintile | 0.87  (0.76 to 0.99) | 0.057 | 0.034 | 857,138 |
| Constant | 0.002  (0.002 to 0.003) | 0.0002 | < 0.001 |  |
| **Model b5** |  |  |  |  |
| 5th quintile  (highest income) | 0.93  (0.85 to 1.02) | 0.043 | 0.120 | 2,210,938 |
| Constant | 0.002  (0.001 to 0.002) | 0.0001 | < 0.001 |  |

* Beneficiary status (BFP=1)

# ¹ All the analytical steps (PS estimation, kernel matching and weighted logistic regressions) were conducted separately for each category of this subgroup.

#

# **Table C - Regression results: Coefficients on unadjusted kernel weighted logistic regressions within subgroups¹ of Cadastro Unico’s decentralized management index (Municipal DMI).**

| **Municipal DMI** | **Weighted Odds Ratio***  **(95% CI)** | **Robust**  **Standard Error** | **p-value** | **N** |
| --- | --- | --- | --- | --- |
| **Model c1** |  |  |  |  |
| 1st quintile (worst) | 0.89  (0.82 to 0.97) | 0.040 | 0.010 | 2,310,241 |
| Constant | 0.002  (0.001 to 0.002) | 0.0001 | < 0.001 |  |
| **Model c2** |  |  |  |  |
| 2nd quintile | 0.89  (0.78 to 1.00) | 0.055 | 0.053 | 1,009,286 |
| Constant | 0.002  (0.002 to 0.003) | 0.0001 | < 0.001 |  |
| **Model c3** |  |  |  |  |
| 3rd quintile | 0.83  (0.72 to 0.96) | 0.061 | 0.010 | 689,665 |
| Constant | 0.003  (0.003 to 0.004) | 0.0002 | < 0.001 |  |
| **Model c4** |  |  |  |  |
| 4th quintile | 0.79  (0.69 to 0.91) | 0.056 | 0.001 | 676,063 |
| Constant | 0.003  (0.002 to 0.003) | 0.0003 | < 0.001 |  |
| **Model c5** |  |  |  |  |
| 5th quintile  (best) | 0.76  (0.66 to 0.88) | 0.055 | < 0.001 | 623,407 |
| Constant | 0.004  (0.003 to 0.005) | 0.0004 | < 0.001 |  |

* Beneficiary status (BFP=1)

# ¹ All the analytical steps (PS estimation, kernel matching and weighted logistic regressions) were conducted separately for each category of this subgroup.

# **Table D - Regression results: Coefficients on unadjusted kernel weighted logistic regressions within subgroups¹ of maternal race/skin color.**

| **Maternal race/**  **skin color** | **Weighted Odds Ratio***  **(95% CI)** | **Robust**  **Standard Error** | **p-value** | **N** |
| --- | --- | --- | --- | --- |
| **Model d1** |  |  |  |  |
| White | 0.90  (0.83 to 0.99) | 0.041 | 0.028 | 1,701,111 |
| Constant | 0.002  (0.001 to 0.002) | 0.0001 | < 0.001 |  |
| **Model d2** |  |  |  |  |
| Mixed/brown (pardo) | 0.81  (0.75 to 0.86) | 0.028 | < 0.001 | 3,311,091 |
| Constant | 0.003  (0.002 to 0.003) | 0.0001 | < 0.001 |  |
| **Model d3** |  |  |  |  |
| Black | 0.74  (0.57 to 0.97) | 0.101 | 0.030 | 239,587 |
| Constant | 0.002  (0.002 to 0.004) | 0.0004 | < 0.001 |  |
| **Model d4** |  |  |  |  |
| Indigenous | 1.00  (0.51 to 1.96) | 0.345 | 0.995 | 35,690 |
| Constant | 0.009  (0.004 to 0.021) | 0.0039 | < 0.001 |  |

* Beneficiary status (BFP=1)

# ¹ All the analytical steps (PS estimation, kernel matching and weighted logistic regressions) were conducted separately for each category of this subgroup.

#

# **Table E - Regression results: Coefficients on unadjusted kernel weighted logistic regressions within subgroups¹ of gestational age at birth.**

| **Gestational age at birth** | **Weighted Odds Ratio***  **(95% CI)** | **Robust**  **Standard Error** | **p-value** | **N** |
| --- | --- | --- | --- | --- |
| **Model e1** |  |  |  |  |
| ≥ 37 weeks | 0.84  (0.79 to 0.89) | 0,025 | < 0.001 | 4,960,905 |
| Constant | 0.002  (0.002 to 0.003) | 0.0001 | < 0.001 |  |
| **Model e2** |  |  |  |  |
| < 37 weeks | 0.78  (0.68 to 0.90) | 0,057 | < 0.001 | 345,266 |
| Constant | 0.004  (0.003 to 0.005) | 0.0005 | < 0.001 |  |

* Beneficiary status (BFP=1)

# ¹All the analytical steps (PS estimation, kernel matching and weighted logistic regressions) were conducted separately for each category of this subgroup.

**Table F - Regression results of interaction analysis. Coefficients on adjusted¹ kernel weighted logistic regression for the association between Bolsa Família Program (BFP) participation and mortality by quintiles of municipal per capita income (MHDI-R) and BFPxMHDI-R interaction term².**

| MHDI-R subgroup | Weighted  Odds Ratio | Robust  Standard error | p-value |
| --- | --- | --- | --- |
| Beneficiary status (BFP=1) | 0.714 | 0.050 | 0.002 |
| BFP x MHDI-R 2nd quintile | 1.052 | 0.099 | 0.999 |
| BFP x MHDI-R 3rd quintile | 1.184 | 0.120 | 0.381 |
| BFP x MHDI-R 4th quintile | 1.214 | 0.116 | 0.170 |
| BFP x MHDI-R 5th quintile | 1.296 | 0.109 | 0.008 |
| LR test* = 57.21 (p-value < 0.001) | | | |

¹ The model was adjusted for prenatal visits, birth weight, gestational age at birth and type of delivery.

² Model equation: BFP + MHDI-R + BFPx MHDI-R

Reference category = Lowest MHDI-R quintile.

* Model without interaction term x the model with interaction term.

**Table G - Regression results of interaction analysis. Coefficients on adjusted¹ kernel weighted logistic regression for the association between Bolsa Família Program (BFP) participation and mortality by quintiles of Cadastro Unico’s decentralized management index (Municipal -DMI) and BFPx M-DMI interaction term².**

| Municipal DMI subgroup | Weighted  Odds Ratio | Robust  Standard error | p-value |
| --- | --- | --- | --- |
| Beneficiary status (BFP=1) | 0.883 | 0.040 | 0.006 |
| BFP x M-DMI 2nd quintile | 0.998 | 0.077 | 0.999 |
| BFP x M-DMI 3rd quintile | 0.939 | 0.080 | 0.999 |
| BFP x M-DMI 4th quintile | 0.891 | 0.074 | 0.666 |
| BFP x M-DMI 5th quintile | 0.863 | 0.073 | 0.332 |
| LR test* = 19.45 (p-value = 0.001) | | | |

¹ The model was adjusted for prenatal visits, birth weight, gestational age at birth and type of delivery.

² Model equation: BFP + M-DMI + BFPx M-DMI

Reference category = 1st quintile (worst).

* Model without interaction term x the model with interaction term.

**Table H - Regression results of interaction analysis. Coefficients on adjusted¹ kernel weighted logistic regression for the association between Bolsa Família Program (BFP) participation and mortality by quintiles of maternal race/skin color and BFP x maternal race/skin color interaction term².**

| Maternal race/skin color subgroup | Weighted  Odds Ratio | Robust  Standard error | p-value |
| --- | --- | --- | --- |
| Beneficiary status (BFP=1) | 0.898 | 0.041 | 0.097 |
| BFP x Mixed/brown | 0.826 | 0.118 | 0.551 |
| BFP x Black | 0.895 | 0.051 | 0.161 |
| BFP x Indigenous | 1.115 | 0.387 | 0.999 |
| LR test* = 19.86 (p-value = 0.002) | | | |

¹ The model was adjusted for prenatal visits, birth weight, gestational age at birth and type of delivery.

² Model equation: BFP + race + BFP x race/skin color

Reference category = White.

* Model without interaction term x the model with interaction term.

**Table I - Regression results of interaction analysis. Coefficients on adjusted¹ kernel weighted logistic regression for the association between Bolsa Família Program (BFP) participation and mortality by quintiles of gestational age at birth and BFP x gestational age at birth interaction term².**

| Gestational age at birth subgroup | Weighted  Odds Ratio | Robust  Standard error | p-value |
| --- | --- | --- | --- |
| Beneficiary status (BFP=1) | 0.840 | 0.025 | < 0.001 |
| BFP x gestational age at birth <37 weeks | 0.934 | 0.074 | 0.389 |
| LR test* = 2.55 (p-value < 0.001) | | | |

¹ The model was adjusted for prenatal visits, birth weight and type of delivery.

² Model equation: BFP + gestational age at birth + BFP x gestational age at birth

Reference category = ≥37 weeks.

* Model without interaction term x the model with interaction term.

# **Table J - Regression results: Coefficients on unadjusted and adjusted inverse probability weighted logistic regressions of BFP participation on mortality between ages one and four**

|  | **Weighted Odds Ratio**  **(95% CI)** | **Robust**  **std. error** | **p-value** | **Weighted Odds Ratio1**  **(95% CI)** | **Robust**  **std. error^1^** | **p-value** |
| --- | --- | --- | --- | --- | --- | --- |
| **Beneficiary status (BFP=1)** | 0.83  (0.78 to 0.87) | 0.0238 | <0.001 | 0.83  (0.78 to 0.87) | 0.0237 | <0.001 |
| **constant** | 0.0029 | 0.0001 |  | 0.0025 | 0.0001 |  |
| **N^2^** | 5,308,989 | | | | |  |

1Model adjusted for number of prenatal visits, birth weight, gestational age at birth, type of delivery

2 Sample size after IPTW

# **Table K - Regression results: Coefficients on unadjusted and adjusted inverse probability weighted logistic regressions within subgroups** **of municipal quintiles of per capita income (MHDI-R).**

| **MHDI-R** | **Unadjusted estimates** | | | | **Adjusted estimates²** | | | |
| --- | --- | --- | --- | --- | --- | --- | --- | --- |
|  | **Weighted Odds Ratio***  **(95% CI)** | **Robust**  **Standard Error** | **p-value** | **N** | **Weighted Odds Ratio***  **(95% CI)** | **Robust**  **Standard Error** | **p-value** | **N** |
| **Model k1** |  |  |  |  |  |  |  |  |
| Constant | 0.005  (0.004 to 0.005) | 0.0003 | < 0.001 | 734,423 | 0.004  (0.004 to 0.005) | 0.0004 | < 0.001 | 714,779 |
| 1st quintile (lowest income) | 0.74  (0.65 to 0.85) | 0.051 | < 0.001 |  | 0.73  (0.65 to 0.85) | 0.052 | < 0.001 |  |
| **Model k2** |  |  |  |  |  |  |  |  |
| Constant | 0.004  (0.003 to 0.004) | 0.0002 | < 0.001 | 823,754 | 0.003  (0.002 to 0.004) | 0.0003 | < 0.001 | 803,236 |
| 2nd quintile | 0.74  (0.65 to 0.85) | 0.050 | < 0.001 |  | 0.74  (0.65 to 0.85) | 0.051 | < 0.001 |  |
| **Model k3** |  |  |  |  |  |  |  |  |
| Constant | 0.003  (0.002 to 0.003) | 0.0002 | < 0.001 | 738,858 | 0.002  (0.002 to 0.003) | 0.0002 | < 0.001 |  |
| 3rd quintile | 0.83  (0.72 to 0.95) | 0.060 | 0.008 |  | 0.83  (0.72 to 0.96) | 0.061 | 0.013 | 722,599 |
| **Model k4** |  |  |  |  |  |  |  |  |
| Constant | 0.003  (0.002 to 0.003) | 0.0002 | < 0.001 | 873,226 | 0.002  (0.002 to 0.003) | 0.0002 | < 0.001 |  |
| 4th quintile | 0.87  (0.76 to 0.99) | 0.057 | 0.036 |  | 0.88  (0.77 to 1.00) | 0.058 | 0.047 | 857,138 |
| **Model k5** |  |  |  |  |  |  |  |  |
| Constant | 0.002  (0.002 to 0.002) | 0.0001 | < 0.001 | 2,255,724 | 0.002  (0.001 to 0.002) | 0.0001 | < 0.001 |  |
| 5th quintile (highest income) | 0.93  (0.85 to 1.01) | 0.042 | 0.096 |  | 0.92  (0.85 to 1.01) | 0.043 | 0.085 | 2,210,938 |

* Beneficiary status (BFP=1)

1 All the analytical steps (PS estimation, ipw estimation and weighted logistic regressions) were conducted separately within each level of these variables.

2 Models adjusted for prenatal visits, birth weight, gestational age at birth, type of delivery.

#

# **Table L - Regression results: Coefficients on unadjusted and adjusted inverse probability weighted logistic regressions within subgroups^1^ of Cadastro Unico’s decentralized management index (Municipal DMI).**

| Municipal DMI | *Unadjusted estimates* | | | | *Adjusted estimates²* | | | |
| --- | --- | --- | --- | --- | --- | --- | --- | --- |
|  | Weighted Odds Ratio*  (95% CI) | Robust  Standard Error | p-value | N | Weighted Odds Ratio*  (95% CI) | Robust  Standard Error | p-value | N |
| **Model l1** |  |  |  |  |  |  |  |  |
| Constant | 0.002  (0.002 to 0.003) | 0.0001 | < 0.001 | 2,358,519 | 0.002  (0.001 to 0.002) | 0.0001 | < 0.001 | 2,310,241 |
| 1st quintile (worst) | 0.86  (0.80 to 0.95) | 0.040 | 0.002 |  | 0.87  (0.80 to 0.95) | 0.040 | 0.002 |  |
| **Model l2** |  |  |  |  |  |  |  |  |
| Constant | 0.003  (0.002 to 0.003) | 0.0002 | < 0.001 | 1,032,697 | 0.002  (0.002 to 0.003) | 0.0002 | < 0.001 | 1,009,286 |
| 2nd quintile | 0.88  (0.78 to 1.00) | 0.055 | 0.037 |  | 0.87  (0.78 to 0.99) | 0.055 | 0,034 |  |
| **Model l3** |  |  |  |  |  |  |  |  |
| Constant | 0.003  (0.003 to 0.004) | 0.0002 | < 0.001 | 705,274 | 0.003  (0.002 to 0.004) | 0.0003 | < 0.001 | 689,665 |
| 3rd quintile | 0.82  (0.71 to 0.95) | 0.059 | 0.007 |  | 0.82  (0.70 to 0.94) | 0.060 | 0,005 |  |
| **Model l4** |  |  |  |  |  |  |  |  |
| Constant | 0.004  (0.003 to 0.004) | 0.0002 | < 0.001 | 692,699 | 0.003  (0.002 to 0.003) | 0.0003 | < 0.001 | 676,063 |
| 4th quintile | 0.80  (0.69 to 0.92) | 0.058 | 0.002 |  | 0.80  (0.69 to 0.93) | 0.059 | 0,003 |  |
| **Model l5** |  |  |  |  |  |  |  |  |
| Constant | 0.004  (0.003 to 0.005) | 0.0003 | < 0.001 |  | 0.004  (0.003 to 0.005) | 0.0004 | < 0.001 |  |
| 5th quintile (best) | 0.77  (0.66 to 0.89) | 0.055 | < 0.001 | 636,768 | 0.77  (0.66 to 0.89) | 0.058 | < 0.001 | 623,407 |

* Beneficiary status (BFP=1)

1 All the analytical steps (PS estimation, ipw estimation and weighted logistic regressions) were conducted separately within each level of these variables.

2 Models adjusted for prenatal visits, birth weight, gestational age at birth, type of delivery.

#

# **Table M - Regression results: Coefficients on unadjusted and adjusted inverse probability weighted logistic regressions within subgroups^1^ of maternal race/skin color.**

| Maternal race/  skin color | *Unadjusted estimates* | | | | *Adjusted estimates²* | | | |
| --- | --- | --- | --- | --- | --- | --- | --- | --- |
|  | Weighted Odds Ratio*  (95% CI) | Robust  Standard Error | p-value | N | Weighted Odds Ratio*  (95% CI) | Robust  Standard Error | p-value | N |
| **Model m1** |  |  |  |  |  |  |  |  |
| Constant | 0.003  (0.002 to 0.003) | 0.0001 | < 0.001 | 1,730,123 | 0.002  (0.002 to 0.003) | 0.0001 | < 0.001 | 1,701,221 |
| White | 0.93  (0.85 to 1.01) | 0.042 | 0.088 |  | 0.92  (0.85 to 1.01) | 0.042 | 0.085 |  |
| **Model m2** |  |  |  |  |  |  |  |  |
| Constant | 0.003  (0.003 to 0.003) | 0.0001 | < 0.001 | 3,390,476 | 0.003  (0.002 to 0.003) | 0.0001 | < 0.001 | 3,311,175 |
| Mixed/brown (parda) | 0.79  (0.73 to 0.84) | 0.028 | < 0.001 |  | 0.79  (0.74 to 0.85) | 0.028 | <0.001 |  |
| **Model m3** |  |  |  |  |  |  |  |  |
| Constant | 0.003  (0.003 to 0.004) | 0.0005 | < 0.001 | 246,054 | 0.003  (0.002 to 0.004) | 0.0005 | < 0.001 | 239,708 |
| Black | 0.69  (0.53 to 0.92) | 0.099 | 0.010 |  | 0.68  (0.51 to 0.90) | 0.098 | 0.007 |  |
| **Model m4** |  |  |  |  |  |  |  |  |
| Constant | 0.006  (0.003 to 0.012) | 0.0020 | < 0.001 | 40,252 | 0.012  (0.003 to 0.037) | 0.0069 | < 0.001 | 38,104 |
| Indigenous | 1.09  (0.55 to 2.17) | 0.383 | 0.800 |  | 0.99  (0.50 to 2.00) | 0.354 | 0.989 |  |

* Beneficiary status (BFP=1)

1 All the analytical steps (PS estimation, ipw estimation and weighted logistic regressions) were conducted separately within each level of these variables.

2 Models adjusted for prenatal visits, birth weight, gestational age at birth, type of delivery.

**Table N - Regression results: Coefficients on unadjusted and adjusted inverse probability weighted logistic regressions within subgroups^1^ of gestational age at birth.**

| Gestational age at birth | Unadjusted estimates | | | | Adjusted estimates² | | | |
| --- | --- | --- | --- | --- | --- | --- | --- | --- |
|  | Weighted Odds Ratio*  (95% CI) | Robust  Standard Error | p-value | N | Weighted Odds Ratio*  (95% CI) | Robust  Standard Error | p-value | N |
| **Model n1** |  |  |  |  |  |  |  |  |
| Constant | 0.003  (0.002 to 0.003) | 0.0001 | < 0.001 | 5,013,232 | 0.002  (0.002 to 0.003) | 0.0001 | < 0.001 | 4,963,543 |
| ≥ 37 weeks | 0.83  (0.78 to 0.88) | 0.025 | <0.001 |  | 0.83  (0.78 to 0.88) | 0.026 | <0.001 |  |
| **Model n2** |  |  |  |  |  |  |  |  |
| Constant | 0.006  (0.005 to 0.007) | 0.0004 | < 0.001 | 350,979 | 0.004  (0.003 to 0.005) | 0.0005 | < 0.001 | 345,446 |
| < 37 weeks | 0.78  (0.67 to 0.90) | 0.060 | <0.001 |  | 0.79  (0.68 to 0.93) | 0.062 | <0.001 |  |

* Beneficiary status (BFP=1)

1 All the analytical steps (PS estimation, ipw estimation and weighted logistic regressions) were conducted separately within each level of these variables.

2 Models adjusted for prenatal visits, birth weight.
