# Supplementary material for: Conditional cash transfer program and child mortality: A cross-sectional analysis nested within the 100 Million Brazilian Cohort
Source: PLoS Med. 2021 Sep 28;18(9):e1003509. doi: 10.1371/journal.pmed.1003509 (PMC8478244; doi:10.1371/journal.pmed.1003509)
Supplement: Attachment — Submitted filename: response to editors and reviewers_May13.pdf [file pmed.1003509.s010.pdf]

Dear Dr. Caitlin Moyer,

Thank you for, once again, giving us the opportunity to submit a revised version of our manuscript. We appreciate the time and effort that you and the reviewer have dedicated to improving the quality of our work. The manuscript has gone through substantial changes, including a new set of analysis and a more in-depth presentation of our findings.

We have been able to incorporate changes to reflect most of the requests and suggestions provided. Below is a point-by-point response to the editors and the reviewer.

Please note that the line numbers in this document are referring to the "marked-up copy" version of the manuscript.

Lastly, in response to requests listed in your decision letter regarding a research protocol and the data availability statement, we inform that we have added our funding proposal project as a prospective analysis plan. Since this study was already completed, we are no longer able to submit a study protocol. We have also provided a statement regarding the availability of our data using the "Data Availability" box at the Additional Information stage of the Editorial Manager system. Please let us know if any other type of information is needed on this regard.

Cordially,
The authors

Requests from the editors:

1. Reviewer Comments: Please fully address the comments of Reviewer 3.

2. Abstract: Line 39, and throughout: Please capitalize Black.
Response: The suggested changes were made.

3. Author summary: Line 62 (and throughout): Please use “low-middle income countries” or similar rather than “developing” countries.
Response: The suggested changes were made.

4. Introduction: Line 127-128: Please revise to avoid causal implications: “To address this gap, we tested the hypothesis that receiving BFP is associated with reduced child mortality…”
Response: The suggested changes were made (lines 132-134).

5. Methods: Please explicitly state in the Methods whether or not your study had a prospectively developed analysis plan. At line 276-278, please refer to the supporting information file containing your protocol (S1_Protocol). Please clarify if the analyses/outcomes described here are prospectively described in the included protocol. In either case, any changes in the analysis-- including those made in response to peer review comments-- should be identified as such in the Methods section of the paper, with rationale.
Response: The suggested changes were made (lines 221-224).

6. Results: Please provide statistics/results to accompany the statements comparing the strength of associations between subgroup associations, for example where associations between BFP and mortality are reported to be stronger for preterm compared to associations for term children: “Considering gestational age, an association between BFP was found for both term (Weighted OR=0.84; CI 95%: 0.79 to 0.89, p<0.0001) and preterm (weighted OR=0.78; CI 95%: 0.68 to 0.90, p<0.0001) children, with stronger association for the latter.” Please also complete this for the DMI quintile and income associations in the previous paragraphs.
Response: We have conducted a formal interaction analysis to address this specific request, following what is common practice in statistics for reporting subgroup effects [1]. Please see lines 310-319 (Methods), 385-393 (Results) and the new Tables S2f-S2i.

7. Discussion: Lines 425-429: Would you please explain further regarding the potential for applying a regression discontinuity design in your study? It would be ideal to conduct this analysis in the current study, if feasible.
Response: Following what was suggested by Reviewer 3, we have removed the aforementioned statement regarding Regression Discontinuity.

8. Discussion: Between the Limitations and Conclusions sections, please include a short section summarizing some implications and next steps for research, clinical practice, and/or public policy;
Response: Implications for policy and future research have been pointed out throughout the text (lines 477-481; 500-505; 521-525).

9. Discussion: Lines 441-443: Please temper causal language throughout, for example, in the following sentence: “...we can also conclude that when properly administered these programmes can increase their already significant impact on child health outcomes.”
Response: The suggested changes were made (lines 559-560).

10. Funding, Competing Interests, Data Sharing: Page 14: Please remove these sections from the main text. This information should be accurately and completely entered in the manuscript submission form.
Response: The suggested changes were made.

11. Patient consent, Page 14: Please remove this section and ensure the relevant participant consent information is noted in the Methods section.
Response: The suggested changes were made (lines 337-338.

12. Table 1: Please present numbers in addition to percentages.
Response: The suggested changes were made. Absolute frequency (N) was added to the unweighted part of the table. The same can not be reported after the weighting stage since the weighted proportions would not be equivalent to a "real" frequency distribution.

13. Table 3: Please define all abbreviations used in the table.
Response: This table was replaced by new ones.

14. Figure 1: Please define all abbreviations in a figure legend (PBF under “exclusions”)
Response: The requested changes were made.

15. RECORD Checklist: Thank you for including the checklist. Please do remove references to page numbers. Locations can be referred to by section, and paragraph within section, such as: “Title Page” or “Methods, 2nd paragraph” for example.
Response: The requested changes were made.

16. Supporting Information Figures and Tables: Please provide a title and legend for each Table and Figure, and please define all abbreviations used in the figures and tables. Please report p values as p<0.001 rather than p<0.000 where applicable. Please define the asterisks (*) in Table S2a.
Response: The requested changes were made.

Comments from the reviewers:

Reviewer #3: I appreciate the efforts of the authors to revise the manuscript, and in particular to tone down the causal language throughout the manuscript. This indeed is appropriate. The revision however points to one new first order concern.

In particular, the results in Table S1c clearly show that for municipalities for which 90% or more of records are submitted, there is no significant relationship between Bolsa and child mortality. This seems to imply that possibly all the results in the paper are driven by sample selection related to missing death records, rather than any real associations between the program and child mortality (as for the subsample for which selection is small, no significant association is found. Most notably, no significative associations are found in municipalities with the full (100%) or almost full (95%) records, and indeed the results there have the opposite sign). On line 431 the authors suggest that loss of records could lead to a small overestimate. This is however not a small overestimate - as the entire relationship becomes insignificant or indeed reversed! This is particularly a concern as a very large share of the observations (3.8 million) come from municipalities with 50% of records missing. At the very least, a bounding exercise based on reasonable assumptions about the missing records would seem important, though given the magnitude of the selection, this is likely to result in very wide confidence intervals, which would unfortunately confirm that little can be said from this incomplete data about the true relationship.

Response: To address this concern, several actions have been taken and we conducted additional analyses.

Below is the distribution of % nameless records submitted to the linkage process by municipality. The dataset that originated this table has the 5,565 Brazilian municipalities (plus one group of "ignored/ missing" municipalities) as the units of analysis. We got to these percentages by dividing the number of nameless death records of children under five years old by the total of deaths of children under five years old in each municipality. As demonstrated, 50% of municipalities had 92% of their nameless death records submitted to linkage, and less than 1% of the municipalities had less than 66% of their nameless records submitted.

```
. sum submetido_1, d

                        (mean) submetido_1
-------------------------------------------------------------
      Percentiles      Smallest
 1%     66.66666             20
 5%         77.5       33.33333
10%        81.25       33.33333       Obs               5,566
25%     86.66666       33.33333       Sum of Wgt.       5,566

50%     92.64957                      Mean           91.48936
                        Largest       Std. Dev.      8.093978
75%     99.06542            100
90%          100            100       Variance       65.51247
95%          100            100       Skewness      -1.403597
99%          100            100       Kurtosis       7.282028
```

It is also important to remember that this loss refers only to the death records that did not have a name in the death certificate, therefore were not eligible for the linkage process. As shown in Table S1b, nameless records correspond to only 3.99% of the total of 1-4 year old death records for Brazil

considering our analysis period (5218 nameless records out of a total of 130858 deaths). It would be difficult to assume, as stated by the reviewer, that:

> "*all the results in the paper are driven by sample selection related to missing death records*",

since we are actually dealing only with losses of an already small percentage of nameless death records, not the whole of the mortality data.

Moreover, our original idea for the previous version of Table S1c was to estimate how allowing different levels of this linkage loss would affect our estimates in each of these groups of municipalities. As stated in former lines 346-351:

> *"Overall, 1277 municipalities had no nameless records lost before linkage, 2185 municipalities had at least 95% of all nameless records submitted, 3488 municipalities had at least 90%, 4517 municipalities had at least 85%, 5147 municipalities had at least 80%, 5386 municipalities had at least 75%, 5557 municipalities had at least 50% of nameless death records submitted to linkage and 10 municipalities had all their nameless death records excluded from the linkage process."*

What we meant by this paragraph was that our groups were cumulative. The 50% group included all observations in municipalities where at least 50% of the nameless records were submitted, that includes all groups up until 100%, contrary to what was stated by the reviewer's comment saying that:

> "*This is particularly a concern as a very large share of the observations (3.8 million) come from municipalities with 50% of records missing*".

The loss was not of 50% of all records, it was of 50% or less out of the nameless records.

Our intention with Table S1c was to ensure complete transparency and report all possible aspects of our findings. Unfortunately, our organization of the data presented in the table seems to have caused a misunderstanding, for which we apologize. To clarify the doubts raised by the reviewer, please find now a revised and restructured version of TableS1c, accompanied by (what we hope to be) a more straightforward explanation of the findings.

In the new version, the number of observations by subgroups of municipalities are classified by non-cumulative (exclusive) cutpoints.

As demonstrated by the new Table S1c, the biggest portion of our sample is from municipalities between the 40.1-90% range (N=3,731,054, equivalent to 70.3% of our total sample of under-five children included in the study n=5,308,989) for which the results are still in accordance to those of our main findings, in terms of magnitude and direction of association. As for the inversion of the expected association in the 100% subgroup, we would like to bring to the reviewer's attention that this subgroup corresponds to only 3% of our study population (N=163,999).

Additionally, we have explored a possible relationship between the percentage or records submitted and our municipal indicators (MHDI-R and DMI). Since one could raise the suspicion that municipal poverty and BFP management were associated with loss of nameless death records, meaning our

subgroup findings were not related to BFP but in fact driven by differential loss of data across the quintiles of MHDI and DMI.

Below are two scatter plots of the distribution of % of nameless records submitted by municipality and MHDI-R (a) and DMI (b), pairwise correlation coefficient of % of records submitted by municipality and MHDI-R and DMI (c).

a) Scatter plot of the distribution of % of records submitted by municipality and MHDI-R

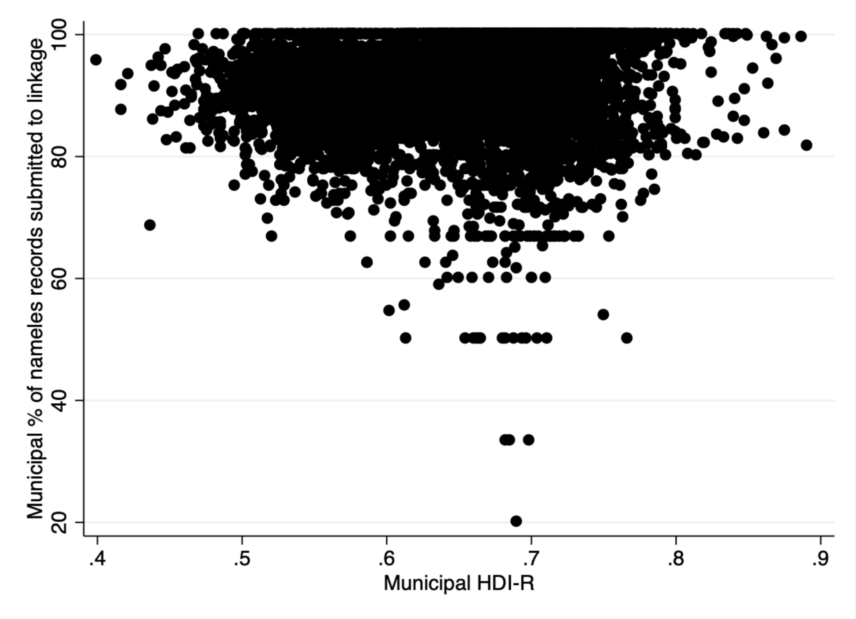

b) Scatter plot of the distribution of % of records submitted by municipality and DMI

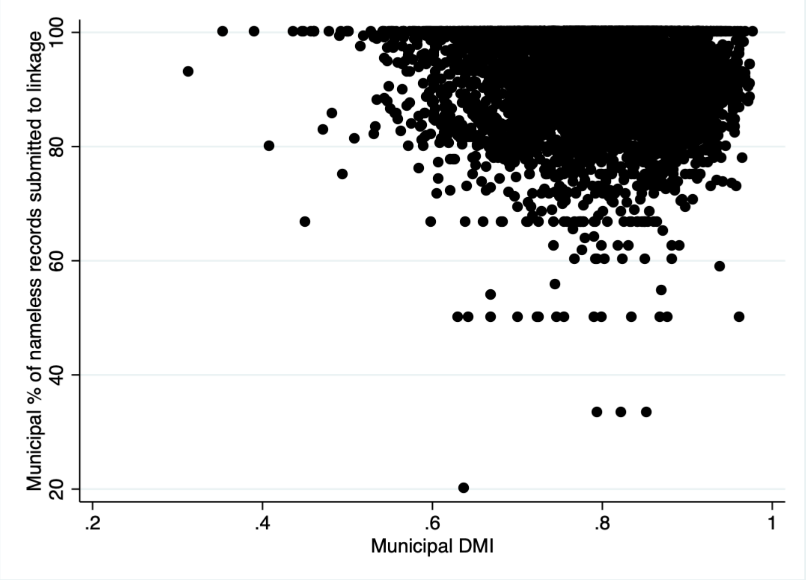

c) Pairwise correlation coefficients and p-values

Variables	(1)	(2)	(3)
(1) Municipal % of records submitted	1.000		
(2) MHDI-R	0.119	1.000	
	(0.000)		
(3) Municipal DMI	-0.068	-0.606	1.000
	(0.000)	(0.000)	

As presented above, the association between the municipal percentage of nameless records submitted to linkage and the municipal indicators used in our subgroup analysis is very weak for the DMI (r=-0.07) and weak for MHDI-R (r=0.11), suggesting those to be factors that are independent of each other and that the subgroup heterogeneity we see within quintiles of DMI and MHDI-R are probably unrelated to differential loss of nameless death records across these strata.

We believe this serves as another test of the validity of findings, and we have rewritten parts of the main text and the supplementary material in order to better present these findings (lines 416-427 in the main text and new paragraphs in the supporting information).

In addition, the revision I continue to have many concerns with this revised manuscript that are similar to the ones raised earlier, but also with the responses to the authors to my earlier comments, which I do not believe fully address the central points I made.

In particular

1) In order to demonstrate the contribution of this paper to the literature, it is crucial not only to review what it is known from the international literature about CCTs and health investments, and what is known about Bolsa Familia in particular, but also specifically to refer to studies in the literature that causally identify the impact of this type of program on child mortality. The current approach in this paper, which refers to the international literature regarding broader health findings, but specifically excludes the papers focused on child mortality (including Barham, 2011 and Okeke and Abubakar, 2020 that I cited earlier), is highly misleading, and frankly hard to understand. I strongly suggest to acknowledge these studies, and to discuss the contribution of the current paper in comparison with them (which appears to be bringing evidence on brazil, at scale and for different subgroups, even if it is not causal).

Response: We again thank the reviewer for his/hers in-depth analysis of our manuscript. We have acknowledged the suggested literature and incorporated a mention to their approach to the discussion of our findings (see lines 115; 534-541).

Indeed, the concern I raised earlier is about the contribution of this paper - not about the plausibility. Given that what we already know from more rigorous designs on how these type of programs affect child mortality, why is showing associational evidence for Bolsa a sufficient contribution for publication in a high impact journal such as Plos Medecine?

Response: We have tried to make clearer statements regarding what we believe to be our contribution to the existing literature (507-515; 539-541). As to whether or not our study brings a sufficient contribution to PLOS Medicine, we believe that to be a decision of the editors.

2) My earlier comments on the drivers of selection into the program are NOT about suggesting a different research question - rather it points out that any study that wants to say something about "effects" (something the authors continue to claim they want to do in their response to my comments) needs to start from a good understanding of the selection criteria, given that the matching estimator proposed by the authors specifically relies on the assumption that it is observable characteristics that drive selection into the program (and that the variables used for that selection are observable to the analysist too).

The references I provided from the economics literature are just some of many that explain (and show empirical support) that in the case of Bolsa Familia, selection is not based on the type of observables the authors account for in the propensity score estimation (which means the assumptions underlying the propensity score estimates are invalid). The concern is in part with manipulation of eligibility - either because of issues with self-reported income, or local political processes. All these capture unobserved factors driving eligibility, and therefore raise serious doubts about the assumptions underlying the matching estimations proposed by the authors (to the extent that it makes it very, very likely that the assumptions are indeed violated.). Here too, ignoring the large literature that has considered this question and instead claiming starting on page 196 that "whether a family receives the program or not is determined by a set of family socio-economic characteristics" is misleading. In this paragraph, a correct discussion on what actually drives selection into the program is important. The statement needs to be revised to give a more comprehensive review of factors affecting receipt of the program.

Given the above, the matching estimator here merely is used as a tool to compare treated and non-treated households that are more similar in observable characteristics, something that may still be useful, but doesn't get you closer to causality. The current wording of the paper is in line with that logic, but being explicit about what the propensity score brings you in this application, and what it doesn't, would seem important to add.

Response: We understand the reviewer's standpoint and have tried to meet their concerns by doing the following:

a- Regarding the limitations of the propensity score approach we stated how we understand the magnitude of such issues, recognizing their impact on the interpretation of our findings (lines 517-525; 534-541).

b- The "local political processes" supposedly involved in Bolsa Familia's eligibility are beyond the scope of our study and have been, as pointed out by the reviewer, a subject matter of other investigations. Although we are aware of this literature we do not have the means to measure if this might have affected our findings nor the grounds to properly discuss whether or not these supposed political processes were in play in our specific subsample. We have tried, however, to improve our clarity in stating the limits of our analytical approach through the use of propensity scores and also

that our methodology is not comparable to that of an experimental or quasi-experimental study (lines 534-541.

c- We have rewritten the referred sentence. It previously read:

> "*whether a family receives the program or not is determined by a set of family socio-economic characteristics*".

Now it reads (lines 214-220):

> "*since whether a family receives the stipend or not is determined by per capita income and a set of family socioeconomic characteristics. Regarding per capita income, concerns about its reliability have been raised, given it is a self-reported measure [27]. To account for the issue of self-selection into the exposure group, we follow a kernel matching approach for the choice of a set of BFP non-beneficiary observations inside the Cidacs' 100 Million Cohort, allowing us to balance the two groups on their observable characteristics and control for possible bias arising from these factors.*"

3) With regard to the point on multiple hypotheses, the concerns stand whether the different hypotheses are tested in the same regression or not. See, for instance, Young (2019), List et al (2019) for related discussions. This is exactly one of the issues that pre-analysis plans aim to address (see Olken 2015). In addition, the main concern here is what all other potential regressions you may have run in the exploratory analysis before setting on the ones reported. All of these tests are additional hypotheses and p-values would need to be adjusted for the fact that those additional hypotheses were tested. I suggest to at least acknowledge this in the manuscript.

Response: We thank the reviewer for explaining in more detail what they meant with this specific request. We would like to clarify that, for variables such as gestational age and maternal race, there was no exploratory classification to be tested, since the subgroups are already settled by the data itself (ie. the groups of race/skin color are simply the ones declared in the questionnaire and the cut points for gestational age are those established by WHO). In that sense, the additional explorations that might have been conducted in the scenario presented by the reviewer are not applicable since the tests by subgroups were quite straightforward. The hypothesis tested are exactly the ones mentioned in our research protocol (see supporting information S4, objective 3 - pages 11 and 12) and the ones already reported in the manuscript.
As for the case of MHDI and DMI, their classification into quintiles was indeed exploratory, and not foreseen by our research protocol, we have now acknowledged this in the manuscript (lines 306-309), explaining that our choice to work with these indicators was guided by the literature (lines 143-154).
Lastly, to improve clarity, we have opted to break down the former Table 3 into separate tables for each subgroup analysis, and we hope to have made it more explicit that these models were all run separately. We have also applied Bonferroni correction to the interaction models reported in the new tables S2f-S2i, since in these cases, the use of interaction terms indeed results in a large number of hypothesis tests.

4) Finally, the specific suggestion made in my earlier comments regarding the matching estimations, following the references cited earlier and common practice in economics, is to

show robustness of results to various alternative matching estimators (1 or 5 nearest neighbour, kernel, etc..). This will help to demonstrate the robustness (or not) of your results.
Response: Following this suggestion, we have conducted additional analysis using IPTW. See lines 288-293 and the new Tables S2j and S2n.

Other comments

Line 218: you motivate not including self-reported income in the propensity score because it would violate the exclusion restriction of the instrumental variable approach but you are not using an IV approach! The assumptions needed for IV are different than for PSM. Matching estimators rely on the assumption that it is possible to account for selection on observables that drive program participation (independently on whether they also affect the outcome or not), so that one can compare 2 groups that according to those observables have equal likelihood of participation (but some for an exogenous reason participated and others didn't). See earlier provided references. Including self-reported income in the estimations gets you closer (in this case) to the assumptions needed for PSM. Therefore, I encourage the authors to show how results change when including self-reported income in the propensity score.

Response: We agree that the income variable would be of great importance to the study of Bolsa Familia, but the data that we have cannot be used for this analysis due to quality issues. We have acknowledged this limitation in the discussion section (lines 517-519).

There is a similar confusion on line 426: IV and RDD are two separate quasi-experimental methods, each with separate sets of assumptions (even if discontinuity in eligibility criteria can sometimes be used as instrument, there are many other types of instruments). In the case of Bolsa, given the evidence of manipulation of income (which would be the running var in RDD estimate), assumptions needed to use a RDD estimation are probably not valid. I hence suggest taking out the reference to RDD.

Response: We have removed the mention to RDD, as requested.

References

List, J., A. Shaikh, and Y. Xu, (2019), "Multiple Hypothesis Testing in Experimental Economics," Experimental Economics (22): 773-793.

Young, A., (2019) "Channelling Fisher: Randomization Tests and the Statistical Insignificance of Seemingly Significant Experimental Results," Quarterly Journal of Economics, 134(2).

Olken, B., (2015), "Promises and Perils of Pre-Analysis Plans," Journal of Economic Perspectives, Vol 29(3): 61-80.

1. Wang R, Lagakos SW, Ware JH, Hunter DJ, Drazen JM. Statistics in medicine--reporting of subgroup analyses in clinical trials. N Engl J Med. 2007;357: 2189–2194.
