# Supplementary material for: Conditional cash transfer program and child mortality: A cross-sectional analysis nested within the 100 Million Brazilian Cohort
Source: PLoS Med. 2021 Sep 28;18(9):e1003509. doi: 10.1371/journal.pmed.1003509 (PMC8478244; doi:10.1371/journal.pmed.1003509)
Supplement: Attachment — Submitted filename: Requests from the editors_DR_NB_RF_MB_March13.docx [file pmed.1003509.s009.docx]

Dear Dr. Caitlin Moyer,

Thank you for giving us the opportunity to submit a revised draft of our manuscript. We appreciate the time and effort that you and the reviewers have dedicated to providing your valuable feedback on our work.

We have been able to incorporate changes to reflect most of the suggestions provided. Below is a point-by-point response to the editors and reviewers’ comments and requests.

Please note that the line numbers in this document are referring to the "marked-up copy" version of the manuscript.

Cordially,

The authors

_________________________________________________________________________

**Requests from the editors:**

*1.Title: Please revise your title according to PLOS Medicine's style. Your title must be nondeclarative and not a question. It should begin with main concept if possible. "Effect of" should be used only if causality can be inferred, i.e., for an RCT. Please place the study design ("A randomized controlled trial," "A retrospective study," "A modelling study," etc.) in the subtitle (ie, after a colon).*

**Response:** We have made the requested changes to the title.

*2.Data availability statement: The Data Availability Statement (DAS) requires revision. Please include the relevant information for requesting access to the data from CIDACS (weblink and/or email contact). The contact cannot be one of the authors of the study. It is not clear if the relevant data are available by accessing the provided link.*

**Response:** We have made the requested changes to the Data Availability Statement (DAS). It now reads as follows: "*All data supporting the findings presented herein were obtained from Centro de Integração de Dados e Conhecimentos para Saúde (CIDACS). Importantly, restrictions apply to access to these data, which contains sensitive information, were licensed for exclusive use in the current study and, due to privacy regulations from the Brazilian Ethics Committee are not openly available. Upon reasonable request and with express permission from CIDACS (mail to* [*cidacs.curadoria@fiocruz.br*](mailto:cidacs.curadoria@fiocruz.br)*) and approval from an ethical committee, controlled access to the data is possible. The dataset is registered under the following DOI handle:* [*https://hdl.handle.net/20.500.12196/CIDACS/65*](https://hdl.handle.net/20.500.12196/CIDACS/65)*, which provides metadata and a register of all versions of the database*".

*3.Throughout: Please include line numbers with the revised version of the manuscript.*

**Response:** Line numbers were added.

*4.Throughout: Please use square brackets for in-text citations, like this [1].*

**Response:** The reference style was changed as requested.

*5.Throughout: Please use “White” rather than “Caucasian”*

**Response:** We have made the requested changes throughout the text.

*6.Abstract: Please structure your abstract using the PLOS Medicine headings (Background, Methods and Findings, Conclusions).*

**Response:** The abstract was changed as requested.

*7.Abstract Background: Provide the context of why the study is important. The final sentence should clearly state the study question.*

**Response:** The abstract was changed as requested.

*8.Abstract: Methods and Findings: Please be sure the abstract describes the population and setting, number of participants, years during which the study took place and main outcome measures.*

**Response:** The abstract was changed as requested.

*9. Abstract: Methods and Findings: Please quantify the main results (with 95% CIs and p values).*

**Response:** The abstract was changed as requested.

*10. Abstract: Methods and Findings: In the last sentence of the Abstract Methods and Findings section, please describe the main limitation(s) of the study's methodology.*

**Response:** The abstract was changed as requested.

*11. Abstract: Conclusions: Please address the study implications without overreaching what can be concluded from the data; the phrase "In this study, we observed ..." may be useful.*

**Response:** The abstract was changed as requested.

*12. Author Summary: At this stage, we ask that you include a short, non-technical Author Summary of your research to make findings accessible to a wide audience that includes both scientists and non-scientists. The Author Summary should immediately follow the Abstract in your revised manuscript. This text is subject to editorial change and should be distinct from the scientific abstract. Please see our author guidelines for more information:* [*https://journals.plos.org/plosmedicine/s/revising-your-manuscript#loc-author-summary*](https://journals.plos.org/plosmedicine/s/revising-your-manuscript#loc-author-summary)

**Response:** We have added the author summary as requested.

*13. Introduction: Please temper claims of primacy: e.g in second last paragraph of introduction: “To the best of our knowledge, this is the first study using nationwide individual level data to analyze the association of BFP with child mortality.”*

**Response:** The sentence was removed.

*14. Methods: Thank you for including the RECORD checklist. Please add the following statement, or similar, to the Methods: "This study is reported as per the REporting of studies Conducted using Observational Routinely-collected Data (RECORD) guideline (S1 Checklist)."*

**Response:** The suggested statement was added (see lines 293-295).

*15. Methods: Did your study have a prospective protocol or analysis plan? Please state this (either way) early in the Methods section.*

*a) If a prospective analysis plan (from your funding proposal, IRB or other ethics committee submission, study protocol, or other planning document written before analyzing the data) was used in designing the study, please include the relevant prospectively written document with your revised manuscript as a Supporting Information file to be published alongside your study, and cite it in the Methods section. A legend for this file should be included at the end of your manuscript.*

*b) If no such document exists, please make sure that the Methods section transparently describes when analyses were planned, and when/why any data-driven changes to analyses took place.*

*c) In either case, changes in the analysis-- including those made in response to peer review comments-- should be identified as such in the Methods section of the paper, with rationale.*

**Response:** We have added the funding proposal as a supporting information file.

*16. Methods: Please move the information pertaining to the study ethical approval and participant consent to the Methods section.*

**Response:** The ethics statement was moved to the Methods section.

*17. Throughout: Please clarify the language used- perhaps “highest income” or “greatest wealth” rather than “richest”*

**Response:** The suggested changes were made throughout the text.

*18. Results: For all findings presented in the text, please provide both the 95% CIs and p values along with the odds ratios. For the adjusted ORs, please also provide results from unadjusted analyses, this can be in a supporting information table.*

**Response:** As requested, 95% CIs and *p*-values were added throughout the text. Unadjusted estimates are now provided in the Supplementary material (Table S2b).

*19. Results: Please avoid the use of causal language (e.g. “effect”) by revising to the following: “Across quintiles of municipal income, associations with BFP ranged from a 28% reduction in the odds of mortality in the poorest municipalities (weighted OR= 0.72; CI 95% [0.62-0.82]) to 13% (weighted OR= 0.87; CI 95% [0.76-0.98]) in the fourth quintile, with no statistically significant association identified with the fifth quintile, indicating a stronger association between BFP and lower risk of mortality for children living in the poorest 20% municipalities of Brazil compared to those in the highest quintile (richest).” Please clarify whether comparisons were done between quintiles, to support the second half of the statement.*

**Response:** We agree that observational studies cannot prove causation. On the other hand, causal questions are not easy to formulate. Hernán [(2018)](https://paperpile.com/c/EDRfuK/7YZ8/?noauthor=1) points out that confounding is a causal concept and if the goal of the observational analysis is purely associational, no adjustment for confounding is necessary. However, the suggested changes in the use of causal language were made throughout the text.

Regarding the subgroup analysis, it is important to mention that all subgroups analysis were based on inverse relationship between program coverage and social and economic indicators in the municipal levels [(Faciroli & Sirqueira, 2017; Souza et al., 2013](https://paperpile.com/c/EDRfuK/MpttC+r55fv); [Araújo et al., 2015; de Abreu Pereira Silva et al., 2012; Mendes, 2018; van Stolk & Patil, 2013](https://paperpile.com/c/EDRfuK/PS6od+wZXVw+1eNNq+MW6Lp); [Rocha, 2011](https://paperpile.com/c/EDRfuK/e9gzo); [Senna et al., 2016)](https://paperpile.com/c/EDRfuK/xBc9Y). Therefore, all results showed a strong association in the lowest stratum levels. Furthermore, as now stated in the methods section (lines 258-271) and in the footnote for tables 3 and S2b, all our analysis steps were conducted separately for each category within DMI, MHDI, Maternal race and gestational age, rather than stratified. This implies that our subgroup analysis should be interpreted as "the estimates of x on y for group1 and the estimates of x on y for group2'' (as one would get with separate regressions). We have rewritten the quoted paragraph to clarify this.

*20. Results: Please clarify whether tests were done comparing DMI quintiles: “The association between BFP and mortality between ages 1-4 was also stronger in municipalities at the highest DMI quintile, ranging from a 12% reduction in the odds of mortality in the lowest quintile (weighted OR=0.88; CI 95% [0.81-0.96]) to 24% in the highest (weighted OR=0.76; CI 95% [0.66-0.88]), suggesting a stronger association of BFP with lower risk of child mortality in municipalities in which the program is best administered.”*

**Response:** Please refer to our answer to comment #19.

*21. Results: Please revise to avoid causal language: “Considering differences in association across individual characteristics, associations between BFP and child mortality were found in all race groups…” or similar.*

**Response:** Aware of the limitations arising from our methodology and issues of data quality, we have made a conscious effort to revise the text and avoid claims of causality throughout the entire manuscript by using the word *association* instead of "impact" or "effect". Following what was discussed in other publications on this matter [(Altman & Krzywinski, 2015; Hennekens & DeMets, 2011)](https://paperpile.com/c/EDRfuK/j9Io+ZRfH), we hope to have made this issue clearer to the future readers of this study.

*22. Results: Please provide the analyses supporting this statement, or clarify if statistical significance is meant here: “The percentage of nameless death records not submitted to linkage at the municipal level were significantly different across Brazil.”*

**Response:** The quoted text was rewritten and now it reads as follows: *"The percentage of nameless death records not submitted to linkage at the municipal level was considerably different across Brazil"* (lines 371-372).

*23. Results: Please clarify this sentence, revising to avoid causal language: “The null value for the 95% and 90% subgroups of municipalities are well within their confidence intervals, indicating no significant effects of the program in these subgroups (see Table 1c - supplementary material 1).”*

**Response:** We have made changes throughout the text to avoid causal language (see response to comment #21). The quoted sentence was changed accordingly (lines 390-393).

*24. Discussion: Please avoid the use of causal language throughout, e.g.”effect on” in the second paragraph: “Because of our large sample, it was also possible to explore heterogeneity in effect of BFP on 1-4 years old mortality across multiple subgroups…”*

**Response:** We have made changes throughout the text to avoid causal language (see response to comment #21). The quoted sentence was changed accordingly (line 413).

*25. Conclusions: Please revise to avoid the use of causal language “Using data from the 100 Million Brazilian Cohort, we conclude that the programme had a significant effect on reducing child mortality, especially in municipalities with low per capita income. Moreover, as the magnitude of the effect grew in municipalities with better indicators of BFP targeting and management, we can also conclude that when properly administered these programmes can increase their already significant impact on child health outcomes.”*

**Response:** We have made changes throughout the text to avoid causal language (see response to comment #21). The quoted sentence was changed accordingly (lines 491-496).

*26. References: Please use the "Vancouver" style for reference formatting, and see our website for other reference guidelines* [*https://journals.plos.org/plosmedicine/s/submission-guidelines#loc-references*](https://journals.plos.org/plosmedicine/s/submission-guidelines#loc-references)

**Response:** We have revised the reference formatting.

*27. Checklist: When completing the RECORD/STROBE items on the checklist, please use section and paragraph numbers, rather than page numbers.*

**Response:** We indicated exact paragraphs in all items where the information was limited to a section of the text. In all other cases, only pages were indicated as the RECORD information was dispersed throughout various sentences (eg. statistical analysis), making it not possible to indicate an exact section or paragraph numbers.

*28. Figures/Tables: Please provide titles and legends for all figures and tables.*

**Response:** We have corrected the figures that did not have an appropriate title or legend. All figures were now uploaded separately from the manuscript and its legends and titles are inserted in the text as instructed by: <https://journals.plos.org/plosmedicine/s/figures#loc-how-to-submit-figures-and-captions>

*29. Figure 1: Please provide a descriptive legend, and please define the abbreviation for PBF.*

**Response:** We have provided a title and corrected the abbreviation.

*30. Figure 2: Please indicate in the legend if these are adjusted or unadjusted OR- and if adjusted please list the variables included in the adjustment. Please provide the unadjusted analyses.*

**Response:** We have corrected the legends for each figure informing the covariate adjustment. Unadjusted estimates are now provided in the Supplementary material (Table S2b).

*31. Table 1: Please define abbreviations in the legend (BFP) and please describe the BFP1-BFP0 in the legend.*

**Response:** We have made the requested changes.

*32. Table 2 and Table 3: Please provide the p values associated with these OR*

**Response:** We have made the requested changes.

*33. Table 3: Please indicate in the legend if these are adjusted OR, and indicate the variables included in the adjustment in the legend.*

**Response:** We have made the requested changes.

*34. Supporting information files: Please include a descriptive title and legend for each figure/table in the Supporting Information files.*

**Response:** We have added the requested information.

*35. Supporting information Table 1C: Please revise the title to avoid causal implications: “Table 1c - Estimates of BFP impact on child mortality…” Please also provide p values, and note factors included for adjustment.*

**Response:** We have changed the table accordingly.

*36. Supporting information Table 2A: Please also provide p values for these associations.*

**Response:** We have added *p-*values as requested.

**Comments from the reviewers:**

***Reviewer #1:*** *It is a relevant manuscript to the public health area, since it addresses the issue related to the access to cash transfer program for low-income Brazilian population and infant mortality. Therefore, it is aligned with the scope of the journal Plos Medicine.*

*The manuscript aimed to investigate the association between receiving Programa Bolsa Familia and the risk of mortality before 5 years old, through a 100 Million Brazilian Cohort whose data were obtained from Cadastro Único (CadÚnico).*

*The manuscript is well written and structured.*

*In the Introduction section, the authors described the present state of scientific literature related to the central theme, pointing out how far the studies have come and the gap to be filled to advance knowledge about the effects of income transfer programs in reducing poverty and mortality of children under 05 years. In this sense, the hypothesis was highlighted.*

*In the Methods section, procedures and analyzes for achieving the objectives were consistently listed. Results and Discussion were equally clear and concise, pointing out the reach and the limits of the study. In the Conclusion section, authors summarized the fundamental findings.*

*Due to its unprecedented nature, as it is the first national study with individual data investigating the effects of a cash transfer program on infant mortality, I recommend the manuscript to be eligible for publication in Plos Medicine Journal, after minor changes listed below:*

*Abstract:*

*"Conclusions: These findings reinforce the evidence that CCTs like BFP..."*

*- I suggest the authors write the full name for CCTs, once it is the first time it appears in the text.*

**Response:** Due to requests from the editors, the abstract was restructured and rewritten, which incurred the removal of the CCT acronym.

*Introduction:*

*- Fourth line: authors might write the full name of MGD 4*

**Response:** We have made the suggested change.

*Tables and Figures:*

*- It is necessary to review the titles of Tables and Figures, as well as to include captions for the acronyms. Tables and figures should be self-explanatory, without requiring the readers to use the text for their understanding.*

**Response:** We have revised all our tables and figures and added captions for the acronyms.

*References:*

*- It would be interesting to update the references, especially those below the year of 2014.*

**Response:** We believe this is an important point, as 41% of our references (19 out of 46) are below the year 2014. However, we are afraid it is not possible to exclude or update most of those references since they refer to important topics such as:

- available studies of the Bolsa Familia Program on child health (references number 7, 8, 11, 12, 13 and 19);
- the only detailed description of the implementation of the Bolsa Familia Program written in English language (reference number 21);
- historical analysis of the Bolsa Familia Program (references number 24 and 25)
- available studies of other CCTs in Latin America (references 40, 42-44)

***Reviewer #2:*** *This cross sectional study aims to test the hypothesis that poverty-alleviating policies can reduce child mortality, by examining the association between the Brazilian Bolsa Familia conditional cash transfer program and child mortality (1-4 years) as well as exploring heterogeneous effects by causes of death, maternal race, gestational age, municipality income level and indexes of quality of BFP management.*

*Comments:*

*The RECORD statement checklist of items, extended from the STROBE statement, that should be reported in observational studies using routinely collected health data is provided within the supplementary documentation.*

*"In fact, Bolsa Família is not randomly assigned. Instead, it is a process of families' 'self-selection' since whether a family receives the stipend or not is determined by a set of family socioeconomic characteristics. To account for the issue of self-selection bias into the exposure group, we follow a kernel matching approach for the choice of a set of PBF non-beneficiary observations inside the Cidacs' 100 Million Cohort that can allow us to compare the two groups. "*

*The authors have undertaken a rigorous analytical approach which supports the minimisation of potential biases in the data.*

*See acronym typo in quote - which happens more than once throughout the manuscript and requires remedying.*

**Response:** We apologize for this mistake and have revised all acronyms throughout the text.

*Overall, this is a well written paper with a clearly explained, detailed, thorough, and technically appropriate methodology.*

*The authors have undertaken suitable subgroup analyses, accounted for bias appropriately, and acknowledged the main limitations in the discussion section for accurate and transparent interpretation of the study outcomes.*

***Reviewer #3:*** *Referee report for "Conditional cash transfer program and child mortality: a study of the 100 Million Brazilian Cohort"*

*Main comments*

*This paper uses an impressive sample bringing data together from different administrative sources to analyze the relationship between exposure to Brazil's conditional cash transfer program and child mortality. As such it revisits a question on which there is existing evidence, but with a larger sample (gathering data from 2006 to 2015) covering 5 million children. While the scale is impressive, the exercise unfortunately cannot say much about the causality of the relationship that is being analyzed. And as there is existing rigorously identified evidence from large scale programs (both from quasi-experimental designs, e.g. Barham 2011 in Mexico, covering more than a 1 million children; and from large-scale experimental designs (Okeke and Abubakar 2020 in Nigeria), it is not clear the contribution is sufficient to warrant publication in PLOS Medicine. These papers also need to be recognized, as well as the wider literature in economics on the impact of CCTs on health*

*investments.*

**Response:** We believe the reviewer raises important points with which we partially agree. However, we would like to clarify a few key aspects. Firstly, throughout our introduction section, in order to frame our contribution more adequately, we made an effort to focus our review on evidence more closely related to the Bolsa Familia Program. The reviewer points out the existence of studies of other CCTs outside Brazil that followed more rigorous approaches based on properly randomized interventions, which is not the case of the Bolsa Familia Program and, although they are studies of great quality, would not be comparable to the evidence that can be provided through BFP data and design.

Secondly, aware of BFP's limitations, even in the face of identification issues which we tried to address by the use of weighted estimators, the plausibility of such a relationship between Bolsa Familia and mortality is widely supported by the health literature on the effect of poverty and other socioeconomic constraints on child health outcomes.

Furthermore, as there is evidence of BFP's effect on improving nutrition [(Paes-Sousa et al., 2011)](https://paperpile.com/c/EDRfuK/wS2e), growth monitoring, vaccinations and number of health checkups [(Shei et al., 2014)](https://paperpile.com/c/EDRfuK/K7y0), all of which are key determinants of child mortality, we believe the fundamentals for the study are warranted. There are methodological constraints to our approach, of which we are aware and made a conscious effort to communicate as transparently as possible, but we believe the plausibleness of the association is sufficiently fundamented.

*The reasons the evidence cannot be interpreted as causal is because the assumptions underlying the Kernel matching estimations are most likely violated in this setting. Matching estimators allow for causal identification only if it is reasonable to assume that selection into the program is based on variables that are observable to the analyst. But we know from a relatively large economics literature on Bolsa Familia that exposure to the program is in part driven by political criteria/processes (e.g. de Janvry et al 2012; Brollo et al 2020; among many others), and in part related to self-reported (and therefore likely strategically biased) income. This is indeed why Bolsa is one of the few CCT programs in Latin America without a straightforward set of impact evaluation results.*

**Response:**

- The reviewer raises important points regarding issues with the program's implementation and rules for assignment. This is a valid question, for which we are actively pursuing the answer in our research program, which involves a series of studies of Bolsa Familia and other social protection policies in Brazil. These questions pertaining to the program itself and its political aspects are being constantly raised in the economics literature, and have been since the very beginning of the program back in 2003. However, in our study, we have focused not on the mechanisms of selection into the policy but on a broader analysis of its possible effect on health outcomes of those who were objectively benefited from/exposed to the CCT, making an effort to control for all observable characteristics available in our data sources, as well as conducting sensibility tests of our findings which have been consistent across several subpopulations. More to the point, although suspicions have been raised regarding the straightforwardness and transparency of the mechanisms of selection into the program and the possible political interferences surrounding the monitoring of the conditionalities, we are interested in the association between being exposed to the program and health outcomes of those who are.

- We have read the suggested references and discussed them in great detail. We do not believe, however, that the points raised by de Janvry et al 2012 and Brollo et al 2020 pose objective reasons against our study design or feasibility. Firstly, our outcome measure of mortality was extracted from a source that is completely external to the program, differently from the ones analyzed in the referred papers (i.e. school attendance reported through the program's own conditionalities data). Therefore, it is highly unlikely that mortality recorded through official health data would be subject to manipulation depending on BFP participation. Second, school attendance can indeed influence the receipt of BFP but externally recorded mortality can not, and there is relatively large health literature on the plausibility of the association between child mortality and CCTs, which we believe warrants the effort we made in exploring the association between BFP and mortality.
- We agree that our findings ca not be portrayed as causal, and any affirmation in that direction would be speculative at this time. We have edited the text to state more clearly that our conclusions are the expression of our findings and that we are very cautious not to make any causal claims from these findings (see lines 469-479).

*Was the sub-group analysis pre-specified? If not, why these cuts of the data and not others? Should we not be concerned with multiple hypotheses testing?*

**Response:** The sub-group analysis consisted of a test of possible heterogeneities of the program's association with child mortality across sub-populations, as proposed by a recent literature review on heterogeneous results of CCTs [(Cooper et al., 2020)](https://paperpile.com/c/EDRfuK/AB3L). The sub-groups were chosen according to our hypothesis and guidance from literature, as presented in the last paragraph of the introduction (lines 141-153). Regarding cut-points, we have worked with the original categories of maternal race and the cut-point for gestational age (<37 weeks) follows the WHO guidelines (<https://www.who.int/news-room/fact-sheets/detail/preterm-birth>), widely used in the health literature. DMI has not been analyzed by any other previous study of BFP and health, so our choice of quintilizing the indicator was exploratory and, by doing so, we chose to also quintilize MHDI for consistency.

Regarding multiple hypothesis testing, we would like to clarify that our subgroup analysis was not conducted simultaneously for all the source variables (e.g. maternal race, gestational age, etc). As multiple hypothesis testing refers to instances in which more than one null hypothesis is tested at the same time, we believe this is not the case as we have run all our models separately for each variable, therefore testing each hypothesis on its own (i.e. one regression model was applied for the analysis of DMI quintiles, another separate model was applied for the analysis of MHDI quintiles and so forth). We have corrected the titles for tables 3 and S2b to clarify this point and also modified the text in lines 260-273.

*Other comments*

*The author references other work on the relationship between Bolsa and child mortality, but claims they do better because they use individual data. It is never particularly clear which key questions they think they can answer better with individual data, that cannot be answered with aggregate data. It would be good to highlight this more specifically.*

**Response:** We have added a paragraph regarding this topic (lines 403-411).

*There is a relatively long literature on using bootstrap methods for matching estimators in economics that needs to be recognized. See Abadie and Imbens (2008, 2016) and many related papers, including on bias-corrected matching estimators (Abadie and Imbens, 2011). Also, given the sensitivity of matching estimators to strong assumptions, it is good practice to show robustness with various alternative estimators.*

**Response:** We have edited the text, and we included two of the suggested references (lines 245-247).

*References*

*Abadie, Alberto and Guido Imbens, 2011. Bias-Corrected Matching Estimators for Average Treatment Effects Journal of Business and Economic Statistics, January 2011, 29(1), 1-11.*

*Abadie, Alberto and Guido Imbens, 2008. On the Failure of the Bootstrap for Matching Estimators Econometrica 76(6), 1537-1557.*

*Abadie, Alberto and Guido Imbens, 2016. Matching on the Estimated Propensity Score, Econometrica, 84(2), 781-807.*

*Barham, Tania, A Healthier Start: The Effect of Conditional Cash Transfers on Neonatal and Infant Mortality in Rural Mexico, Journal of Development Economics, 2011, 94(1), 74-85*

*Brollo, F K Kaufmann, E La Ferrara, 2020. The political economy of program enforcement: Evidence from Brazil Journal of the European Economic Association 18 (2), 750-791*

*de Janvry, Alain, Frederico Finan and Elisabeth Sadoulet, 2012. Local Electoral Accountability and Decentralized Program Performance, with Review of Economics and Statistics, 94(3): 672-685.*

*Okeke Edward and Isa S. Abubakar, 2020. "Healthcare at the beginning of life and child survival: Evidence from a cash transfer experiment in Nigeria" Journal of Development Economics, 143: 102426*

**Cited References:**

[Altman, N., & Krzywinski, M. (2015). Association, correlation and causation. *Nature Methods*, *12*(10), 899–900.](http://paperpile.com/b/EDRfuK/j9Io)

[Araújo, F. R. de, Araújo, M. A. D. de, Souza, F. J. V. de, Santos, D. F., & Santana, M. B. (2015). Uma avaliação do Índice de Gestão Descentralizada do Programa Bolsa Família. *Revista ADM* , *49*(2), 367–393.](http://paperpile.com/b/EDRfuK/1eNNq)

[Cooper, J. E., Benmarhnia, T., Koski, A., & King, N. B. (2020). Cash transfer programs have differential effects on health: A review of the literature from low and middle-income countries. *Social Science & Medicine*, *247*, 112806.](http://paperpile.com/b/EDRfuK/AB3L)

[de Abreu Pereira Silva, A., Ferreira, M. A. M., & Monteiro, D. A. A. (2012). Desempenho na Gestão Pública do Programa Bolsa Família sob a Perspectiva de Análise do Índice de Gestão Descentralizada (IGD). *Desenvolvimento em Questão*, *10*(21), 211–241.](http://paperpile.com/b/EDRfuK/MW6Lp)

[Faciroli, J., & Sirqueira, T. F. M. (2017). Análise espacial da taxa de cobertura do Programa Bolsa Família para os municípios brasileiros. *Desenvolvimento Regional em debate: DRd*, *7*(2), 118–137.](http://paperpile.com/b/EDRfuK/r55fv)

[Hennekens, C. H., & DeMets, D. (2011). Statistical association and causation: contributions of different types of evidence. *JAMA: The Journal of the American Medical Association*, *305*(11), 1134–1135.](http://paperpile.com/b/EDRfuK/ZRfH)

[Hernán, M. A. (2018). The C-Word: Scientific Euphemisms Do Not Improve Causal Inference From Observational Data. *American Journal of Public Health*, *108*(5), 616–619.](http://paperpile.com/b/EDRfuK/7YZ8)

[Mendes, L. H. (2018). *Avaliação do Desempenho do Programa Bolsa Família nos Municípios do Nordeste Brasileiro nos anos de 2008 e 2012 com base no Índice de Gestão Descentralizada (IGD)* [Universidade Federal do Rio Grande do Norte].](http://paperpile.com/b/EDRfuK/wZXVw) <http://monografias.ufrn.br/jspui/handle/123456789/6811>

[Paes-Sousa, R., Santos, L. M. P., & Miazaki, É. S. (2011). Effects of a conditional cash transfer programme on child nutrition in Brazil. *Bulletin of the World Health Organization*, *89*(7), 496–503.](http://paperpile.com/b/EDRfuK/wS2e)

[Rocha, S. (2011). O programa Bolsa Família: evolução e efeitos sobre a pobreza. *Economies et societes*, *20*(1), 113–139.](http://paperpile.com/b/EDRfuK/e9gzo)

[Senna, M. de C. M., Brandão, A. A., & Dalt, S. D. (2016). Family Allowance Program and the follow-up of the conditions related to the health field. *Serviço Social & Sociedade*, *125*, 148–166.](http://paperpile.com/b/EDRfuK/xBc9Y)

[Shei, A., Costa, F., Reis, M. G., & Ko, A. I. (2014). The impact of Brazil’s Bolsa Família conditional cash transfer program on children's health care utilization and health outcomes. *BMC International Health and Human Rights*, *14*, 10.](http://paperpile.com/b/EDRfuK/K7y0)

[Souza, A. P. F. de, Duarte, J., Neves, J. de A. S., Oliveira, P. P. de, & Gadelha, S. R. de B. (2013). *Uma investigação sobre a focalização do Programa Bolsa Família e seus determinantes imediatos*.](http://paperpile.com/b/EDRfuK/MpttC) <http://hdl.handle.net/10438/11345>

[van Stolk, C., & Patil, S. (2013). *Understanding the quality of implementation in Bolsa Familia*. https://doi.org/](http://paperpile.com/b/EDRfuK/PS6od)[10.35648/20.500.12413/11781/ii013](http://dx.doi.org/10.35648/20.500.12413/11781/ii013)
