# Supplementary material for: Conditional cash transfer program and child mortality: A cross-sectional analysis nested within the 100 Million Brazilian Cohort
Source: PLoS Med. 2021 Sep 28;18(9):e1003509. doi: 10.1371/journal.pmed.1003509 (PMC8478244; doi:10.1371/journal.pmed.1003509)
Supplement: Attachment — Submitted filename: response letter July20_MB.docx [file pmed.1003509.s011.docx]

Salvador, July 20, 2021

Dear Dr. Caitlin Moyer,

Thank you for giving us another opportunity to submit a revised draft of our manuscript.

We deeply appreciate the time and effort that the PLOS Editorial team of editors and reviewers has dedicated to providing such valuable feedback on our manuscript.

We were able to incorporate changes to reflect all of the suggestions provided in this round. They are highlighted as tracked changes within the "marked-up copy" of the manuscript.

Below is a point-by-point response to the editors’ comments and concerns. Please note that the line numbers in this document refer to the "marked-up" file.

Cordially,

The authors

-----

Requests from the editors:

1. From the academic editor: We request that you please more thoroughly acknowledge in the manuscript the limitations pointed out by reviewer 3, regarding the missing data (records not available for linkage) and the potential for this to impact the findings and conclusions of the study.

**Response:** We have made changes to the abstract (lines 93-96) and the limitations (lines 568-582) sections in order to address this request.

2. Title: Please revise your title according to PLOS Medicine's style. We suggest: “Conditional cash transfer program and child mortality: A cross-sectional analysis nested within the 100 Million Brazilian Cohort”

**Response:** The suggested change was done accordingly.

3. Abstract: Line 24: We suggest changing to (1-4 years of age) or similar.

**Response:** The suggested change was done accordingly.

4. : Line 38 and line 43: Please report this as p<0.001.

**Response:** The suggested change was done accordingly.

5. Abstract: Line 45: Please be more specific if possible regarding the “small over-estimate” attributable to the loss of nameless death records before linkage.

**Response:** We revised the sentence accordingly (see lines 45-46).

6. Author summary: Why was this study done? In the first point, we suggest “reaching” rather than “completing” if appropriate.

**Response:** The suggested change was done accordingly.

7. Introduction: Line 112: Please replace “developed countries” with high-income countries.

**Response:** The suggested change was done accordingly.

8. Methods: Line 248: Please list the perinatal conditions adjusted for here (although they are also noted in Table 4).

**Response:** Revised accordingly (lines 262-263).

9. Results: Throughout the Results section, please report p<0.0001 as p<0.001.

**Response:** The suggested change was done accordingly.

10. Results: Please include more description and interpretation of the results presented in supporting information tables S1a, S1b, and S1c.

**Response:** Please note that the tables numbers changed as a consequence of request #17 of the editors. References to tables S2a, S2b, and S2c were added to the results (lines 412, 423, 444) and limitations section (line 535).

11. Discussion: Limitations Lines 477-484: In the sentence describing the loss of nameless records (“...loss of nameless records before linkage seems to be resulting in a possible over-estimation of the association…”) it would be helpful here, and in the appropriate section of the Results, to quantify or define the over-estimation. You mention in this paragraph that the majority of observations do come from municipalities where associations were consistent with the sample as a whole. However, as brought up previously by Reviewer 3, we request that you more thoroughly acknowledge the limitation of missing data and the impact on results, and discuss the potential for this to confound the conclusions.

**Response:** Revised accordingly (changes applied to the second to last paragraph of the discussion section).

12. Figure 1: Please include a descriptive legend for this figure.

**Response:** The suggested change was done accordingly. The legend was added below the title for figure 1.

13. Figure 2: Please define MHDI-R and DMI in the legend. We suggest changing the y axis to something more descriptive. If possible, please present all panels on the same range of y axis values, or note the different y axis scales in the legend.

**Response:** Revised accordingly.

14. Table 3: Please define the abbreviation (MHDI-R) and please describe in the legend the factors that distinguish each of the models.

**Response:** The suggested change was done accordingly, with the abbreviation for MHDI-R added within parenthesis. As for the description of the models, there aren't any factors that distinguish them, considering they were only run separately for each MHDI-R quintile but were all adjusted considering the same covariates. It is described in the table footnote.

15. Table 4: Please define the abbreviation DMI in the legend, and please describe the factors that distinguish each of the models.

**Response:** The suggested change was done accordingly, with the abbreviation for DMI added within parenthesis. As for the description of the models, there aren't any factors that distinguish them, considering they were only run separately for each DMI quintile but were all adjusted considering the same covariates. This is described in the table footnote.

16. Tables 5 and 6: Please describe the factors that distinguish each of the models.

**Response:** As it was done in tables 3 and 4, there aren't any specific factors distinguishing these models. They were adjusted separately within each category/level/quintile of the variables specified in the title and in the caption/legend.

17. Study Protocol: We suggest renaming the file to “S1_Protocol” and double checking to be sure all information is intended to be included (e.g. the contact information on the first page, and the investigator CVs at the end of the document).

.

**Response:** We thank the editors for identifying this issue. As requested, we have renamed the file, omitted personal information from the protocol, and removed the CVs in the last pages.

18. RECORD Checklist: Please replace the location of the Background rationale (currently noted as pages 3 and 4) with the appropriate section/paragraph of the main text (for example, within the Introduction).

**Response:** Revised accordingly.

19. Figure S1a: Please include a legend, with all abbreviations used within the figure defined.

**Response:** Revised accordingly. The legend was added below the title for the figure.

20. Figure S2: We suggest revising “treated” and “untreated” to “beneficiaries” and “non-beneficiaries” respectively.

**Response:** Revised accordingly
