# Supplementary material for: Conditional cash transfer program and child mortality: A cross-sectional analysis nested within the 100 Million Brazilian Cohort
Source: PLoS Med. 2021 Sep 28;18(9):e1003509. doi: 10.1371/journal.pmed.1003509 (PMC8478244; doi:10.1371/journal.pmed.1003509)
Supplement: Attachment — Submitted filename: response letter_Aug4.docx [file pmed.1003509.s012.docx]

**Salvador, August 10 2021**

Dear Dr. Caitlin Moyer,

We were able to incorporate changes to reflect all of the editors' requests. They are highlighted as tracked changes within the "marked-up copy" of the manuscript.

Below is a point-by-point response to the editors’ requests

Cordially,

The authors

**--**

**Requests from Editors:**

1. Data availability statement: Please check that the DOI for the data is accurate/working. At this time, following the link leads to an error message (“Error: Cannot connect to server”).

**Response:** The dataverse website was under maintenance during the past week but it has been fixed now. The DOI handle is back online at <https://hdl.handle.net/20.500.12196/CIDACS/65>

2. Throughout: Please carefully check the grammar and language throughout the text for errors.

**Response:** Revised accordingly.

3. Abstract: Introduction: At line 25, we suggest replacing “gestational age” with “preterm birth” if this helps to clarify the objective. At line 24-26, we suggest revising to “...also examining how this association differs by maternal race/skin color, preterm birth, municipality income level, and indexes of quality of BFP management.” or similar.

**Response:** Changed accordingly.

4. Abstract: Methods and Findings: Line 38: Please remove the extra space in the reported p value.

**Response:** Changed accordingly.

5. Abstract: Methods and Findings: Line 38-39: Please also provide weighted OR, 95% CI and p values for the BFP-mortality associations for preterm birth and children of Black mothers, as is reported for municipality income status and BFP management status.

**Response:** Changed accordingly.

6. Abstract: Methods and Findings: Line 44-45: Please remove the word “small” and revise the sentence to: “Furthermore, sensitivity analyses showed that loss of nameless death records before linkage may have resulted in an over-estimate of the measured associations between BFP and mortality…”

**Response:** Changed accordingly.

7. Author summary: Why was this study done? In the first point, we suggest adding “globally” if this is meant. In the third bullet point, we suggest revising to: “...the world’s largest conditional cash transfer program)...” if this is accurate.

**Response:** Changed accordingly.

8. Author summary: What did the researchers do and find? In the second point, we suggest revising to: “...gestational age at birth…” or similar.

**Response:** Changed accordingly.

9. Author summary: What do these findings mean? In the first point, please revise to “Our findings are consistent with previous studies…” or similar. We suggest revising the wording of the second point to: “The greater association among more vulnerable groups of children suggests conditional cash transfers may help to promote equity, with stronger results among those more in need.”

**Response:** Changed accordingly.

10. Introduction: In the first paragraph, please make sure that all statements/reported statistics are appropriately referenced with citations.

**Response:** Changed accordingly. A new reference was added.

11. Introduction: Line 112: Please revise to: “...they are also present in countries including....”

**Response:** Changed accordingly.

12. Introduction: Line 115: We suggest changing “speedily reached” to “...rapidly implemented throughout the country…”

**Response:** Changed accordingly.

13. Introduction: Line 139: Please clarify to “gestational age at birth” if this is meant. It may be more straightforward throughout to refer to preterm birth status as opposed to gestational age, as gestational age was categorized into preterm or term births in your analyses.

**Response:** Changed accordingly. We have opted to use "gestational age at birth" throughout.

14. Introduction: Line 139-140: Please move this sentence to the Methods, where you describe your analysis plan: “The subgroup analysis was established by our research protocol (see S4. Research Protocol, objective 3).” - this seems to be approximately lines 211-214.

**Response:** Changed accordingly.

15. Methods: Line 164: Please clarify which supporting information file you are referring to with “supplementary material 1” as this is not clear from the supporting information file names.

**Response:** Changed accordingly.

16. Methods: Line 198: We suggest changing “before the child’s 5th anniversary…” to “prior to the child reaching 5 years of age” or similar.

**Response:** Changed accordingly.

17. Methods: 203: Please provide a more complete reference (or a numbered reference from the References list) for “Bolsa Família. Perguntas Frequentes” - we note that the embedded link does not work.

**Response:** The reference is number #17, for which the correspondent website is <http://mds.gov.br/acesso-a-informacao/perguntas-frequentes/bolsa-familia/beneficios/beneficiario/beneficio-de-superacao-da-extrema-pobreza>, and it is included in the References list.

18. Methods: Line 223: Please note how race/ethnicity was defined/reported and by whom?

**Response:** Changed accordingly.

19. Methods: Line 261: We suggest using “As outlined in our research protocol…” rather than “foreseen” in this sentence.

**Response:** Following the editor's request #17, this sentence was replaced.

20. Methods: Line 287: Please group all of these references within one set of brackets, if appropriate ([20,21,22-25,26,27]).

**Response:** Changed accordingly.

21. Methods: Line 293: Please change “will be based” to “was based” in this sentence.

**Response:** Changed accordingly.

22. Results: Line 370-372: Please avoid using italics for emphasis.

**Response:** Changed accordingly.

23. Discussion: Line 409: Please revise the first sentence to “We found that receiving Bolsa Família is significantly associated with a reduced probability of deaths…”

**Response:** Changed accordingly.

24. Discussion: Line 412: Please revise the sentence to “These findings are consistent with previous studies…”

**Response:** Changed accordingly.

25. Discussion: Line 417: Please revise the sentence to “We believe that interpreting individual-level findings…” or similar.

**Response:** Changed accordingly.

26. Discussion: Line 431: Please revise to “...an in access to health care in Brazil…” if this is meant.

**Response:** Changed accordingly.

27. Discussion: Line 435: Please change “premature” to “preterm” in this sentence.

**Response:** Changed accordingly.

28. Discussion: Line 461: Please add “...to the best of our knowledge” to the end of the sentence, or similar: “...making this the largest study on CCTs and child mortality to date.”

**Response:** Changed accordingly.

29. Discussion: Line 466: Please clarify the heterogeneities that you are referring to here: “We have also accounted for heterogeneities not yet assessed regarding the association of BFP with child health outcomes.”

**Response:** Changed accordingly.

30. Discussion: Line 471: Please change “once” to “because” or similarly clarify what is meant here.

**Response:** Changed accordingly.

31. Discussion: Line 485: Please remove the hyphen from “evidence-based” in this sentence, it seems it should read “...providing evidence based on a large population…”

**Response:** Changed accordingly.

32. Discussion: Line 486-490: We suggest revising to: “Limitations also arise from the linkage process, as loss of nameless records before linkage may have resulted in a possible over-estimation of the association between the program and child mortality, since we observed that in the municipalities without such losses, the direction of the measured association changed, and the lower bound of the confidence interval is very close to the null value of 1.00.”

**Response:** Changed accordingly.

33. Discussion: Line 500: Please change “superior to” to “greater than 40%” in this sentence.

**Response:** Changed accordingly.

34. References: Please check if reference 29 and 30 are duplicates.

**Response:** Changed accordingly.

35. Table 2: Please include the definition for the abbreviation BFP in the legend or elsewhere in the table.

**Response:** Definition added.

36. Figure 2: We suggest including an X axis label for panel C such as “Maternal race” or similar, and revising “Whites” to “White” and “Blacks” to “Black” along the labels. For panel D, we suggest including a note in the legend that Term is defined as greater than or equal to 37 weeks gestation, and Preterm is defined as less than 37 weeks gestation.

**Response:** Changed accordingly.

37. Supporting information: The purpose of the file “Export_20210505_25908PM” is unclear, we suggest removing.

**Response:** The file was removed.

38. Supporting information figure S3a: Please define the abbreviation PBF / please clarify if this should be BFP.

**Response:** Changed accordingly.

39. Tables S2a and S2b: Please check if the commas should be decimal points, “88,00” for example.

**Response:** Changed accordingly.

40. Supporting information file: It might be helpful to separate this document into “S2 Supporting information” and “S3 Supporting Information” to make access easier.

**Response:** Suggestion accepted. Two separate documents were created.
